# Further insights into the Fe(ii) reduction of 2-line ferrihydrite: a semi *in situ* and *in situ* TEM study†

**DOI:** 10.1039/d0na00643b

**Published:** 2020-09-30

**Authors:** Mario Alberto Gomez, Ruonan Jiang, Miao Song, Dongsheng Li, Alan Scott Lea, Xu Ma, Haibo Wang, Xiuling Yin, Shaofeng Wang, Yongfeng Jia

**Affiliations:** Liaoning Engineering Research Center for Treatment and Recycling of Industrially Discharged Heavy Metals, Shenyang University of Chemical Technology Shenyang Liaoning 110142 China mario.gomez@syuct.edu.cn +86 15093716277 +86 15140014967; Department of Geological Sciences, University of Saskatchewan Saskatoon Saskatchewan S7N 5E2 Canada; Physical and Computational Science Directorate, Pacific Northwest National Laboratory Richland Washington 99352 USA Dongsheng.Li2@pnnl.gov +1 5093716242 +1 5093716277; Environmental and Biological Sciences Directorate, Pacific Northwest National Laboratory Richland Washington 99352 USA; Key Laboratory of Pollution Ecology and Environmental Engineering, Institute of Applied Ecology, Chinese Academy of Sciences Shenyang 110016 Liaoning China

## Abstract

The biotic or abiotic reduction of nano-crystalline 2-line ferrihydrite (2-line FH) into more thermodynamically stable phases such as lepidocrocite-LP, goethite-GT, magnetite-MG, and hematite-HT plays an important role in the geochemical cycling of elements and nutrients in aqueous systems. In our study, we employed the use of *in situ* liquid cell (LC) and semi *in situ* analysis in an environmental TEM to gain further insights at the micro/nano-scale into the reaction mechanisms by which Fe(ii)_(aq)_ catalyzes 2-line FH. We visually observed for the first time the following intermediate steps: (1) formation of round and wire-shaped precursor nano-particles arising only from Fe(ii)_(aq)_, (2) two distinct dissolution mechanisms for 2 line-FH (*i.e.* reduction of size and density as well as breakage through smaller nano-particles), (3) lack of complete dissolution of 2-line FH (*i.e.* “induction-period”), (4) an amorphous phase growth (“reactive-FH/labile Fe(iii) phase”) on 2 line-FH, (5) deposition of amorphous nano-particles on the surface of 2 line-FH and (6) assemblage of elongated crystalline lamellae to form tabular LP crystals. Furthermore, we observed phenomena consistent with the movement of adsorbate ions from solution onto the surface of a Fe(iii)-oxy/hydroxide crystal. Thus our work here reveals that the catalytic transformation of 2-line FH by Fe(ii)_(aq)_ at the micro/nano scale doesn't simply occur *via* dissolution–reprecipitation or surface nucleation–solid state conversion mechanisms. Rather, as we demonstrate here, it is an intricate chemical process that goes through a series of intermediate steps not visible through conventional lab or synchrotron bulk techniques. However, such intermediate steps may affect the environmental fate, bioavailability, and transport of elements of such nano-particles in aqueous environments.

## Abstract 2

The catalytic reduction of nano-crystalline 2-line ferrihydrite with Fe(ii)_(aq)_ doesn't occur *via* direct pathways but rather through new intermediate steps.

## Graphical abstract

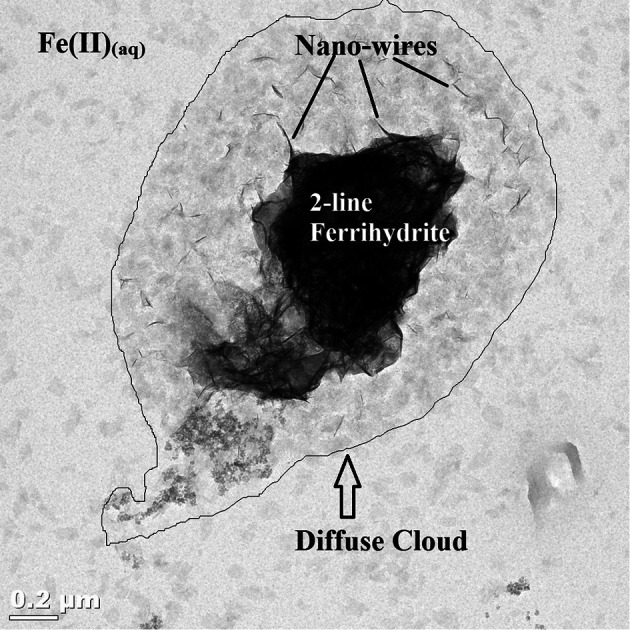

## 1. Introduction

Iron(iii) oxy-hydroxides are some of the most common natural mineral phases that occur in the Earth's crust (*e.g.* soils, volcanic rocks) as well as aquatic settings of ground, surface, and marine environments.^1–6^

2-line FH is a metastable iron(iii) oxy-hydroxide nano-mineral that is commonly found in natural as well as anthropogenic settings.^1,4,7–9^ Due to its widespread occurrence, large surface area, and adsorption capacity, 2-line FH is an extremely important nano-mineral phase. It's often used in the removal and stabilization (under ideal selective conditions) of a range of toxic elements in natural and man-made environments through adsorption and co-precipitation.^1,2,9–12^

The biotic reduction of iron(iii) oxy-hydroxides such as 2-line FH by Fe-reducing bacteria plays an important role as an electron shuttle in various redox processes that control the biogeochemical cycling of transition and metalloid elements (*e.g.* Fe, As, Se, U, Mn) as well as organic matter/nutrients (*e.g.* C, N, and P) in natural and anthropogenic systems.^13–19^ Under anoxic to sub-oxic, non-sulphide rich environments, microbial respiration leads to the generation of Fe(ii)_(aq)_ which can react with iron(iii) oxy-hydroxides to form more thermodynamically stable phases or induce recrystallization and growth within hours to days.^13,20–23^ For bacterial mediated reduction of 2-line FH, various reaction mechanisms have been proposed that include: (1) electron transfer through direct contact between the cell wall and FH surface, (2) electron shuttling of soluble chelating agents such as humic acid, quinones, and flavins with a “labile Fe(iii) phase/reactive-FH” and/or 2-line FH and (3) through extracellular nano-wires.^13,17,24^

In the case of the abiotic reduction of 2-line FH with Fe(ii)_(aq)_, it has been generally agreed upon that the 2-line FH transformation to more thermodynamically stable phases (LP, GT, MG, and HT) is dependent upon several experimental variables. For example, the initial concentration of Fe(ii)_(aq)_, pH, temperature, the presence of solid associated Fe(ii)_(aq)_-FH, soluble ligands (*e.g.* CO_3_, SO_4_, Cl), and co-existing ions (*e.g.* Pb, Mg, Co, Ni, Zn, Si).^20,21,25–28^ Many previous works have indicated that the process of Fe(ii)-2-line FH induced recrystallization mainly occurs *via* dissolution-reprecipitation for the formation of LP and GT. Meanwhile, surface nucleation, solid-state conversion, and growth favours MG precipitation.^13,20,21,29^ Though more detailed research has indicated that the major reaction steps in current proposed conceptual models comprise of (1) the adsorption of Fe(ii)_(aq)_ onto 2-line FH, (2) immediate oxidation of Fe(ii)-2-line FH to Fe(iii) with electron exchange to other Fe(iii) lattice atoms, (3) atom exchange at the interface of the redox couple in step 2, (4) electron conduction through the solid, (5) the production of a “reactive surface FH/labile Fe(iii)”, (6) reductive release of Fe(ii)_(aq)_, (7) continuing cycle of 1–6 until LP, GT, MG or HT are precipitated on the surface of the “reactive-FH/labile Fe(iii) phase” and finally (8) complete bulk transformation of the initial 2-line FH to LP, GT, MG or HT that depends upon the experimental conditions.^20,21,23,29,30^ Evidence for these more detailed steps in conceptual models have come from decades of research *via* the use of solution (*e.g.* ICP-OES/MS, AAS, UV-VIS, isotope labelling, adsorption models), solid (*e.g.* EPR, XPS, XAS, Mössbauer, XRD, X-ray reflectivity, STM, STXM, AFM, *ex situ* TEM, IR/Raman) and computational analysis.^20,21,23,29–31^

Development of LC TEM and the environmental TEM, as well as their use over the last couple of decades, has revolutionized the way we have understood nucleation pathways of nano-particles; their coalescence; nano-crystal growth and dissolution; shape and crystal face evolution; nano-particle solution dynamics; electrochemical reactions and some biological processes.^32–39^ However, to the best of the author's knowledge, the biotic or abiotic reduction of FH (or other iron(iii) oxy-hydroxides) with Fe(ii)_(aq)_*via* the use of LC TEM has not yet been investigated. Some hydrogen gas reduction of FH to MG *via in situ* TEM has been reported by Hummingbird Scientific^40^ but to date, no literature exists for such reactions in liquids *via* semi *in situ* or *in situ* in LC TEM.

Therefore, as a result of the absence of knowledge that could be gained *via* the dynamic high-spatial-resolution of LC TEM, we decided to undertake (1) *in situ* and semi *in situ* analysis at pH ∼ 6 with 0.2 mM and 10 mM Fe(ii)_(aq)_ and (2) *in situ* analysis at pH 8 and 10 mM Fe(ii)_(aq)_ of the well documented abiotic Fe(ii)_(aq)_ reduction with 2-line FH (Fig. S0†). This was done to confirm some hypothesized, but not yet visually observed events in the literature as well as to provide new insights and information at the micro/nano-scale that bulk characterization techniques cannot provide. Furthermore, based on the gathered information, a new revised conceptual model is proposed.

## 2. Experimental section

### 2.1 Synthesis and reduction experiments

All reagents used in this work (Fe_2_(SO_4_)_3_·5H_2_O, NaOH, FeSO_4_·7H_2_O) were of ACS grade and purchased from Sinopharm Chemical Reagent Co. Ltd. All reagents had ≤0.1% impurities and as such are not expected to have any significant effect on our reactions. In all cases, deionized water (DI) and deoxygenated DI water were used for the reaction experiments. The DI water used for bulk tests done to verify semi *in situ* and *in situ* TEM results was deoxygenated by bubbling nitrogen gas at high pressure in the DI water for ∼3 hours before placing into our anaerobic glove box-1 (Etelux Lab 2000 with O_2(g)_ levels ranging from 0.1 ppm to 10 ppm) to stand overnight before use. The DI water used for the semi *in situ* and *in situ* TEM tests was deoxygenated by boiling water at 100 °C for 2 h while purging N_2(g)_ at high pressure. After this, the deoxygenated water was stored in glove box-2 (Innovative Technology Inc. with O_2(g)_ levels of ∼0.8 ppm) for ∼48 h before use.

The synthesis of the 2-line FH was done according to a modified method by Cornell and Schwertmann^2^ in which a ferric sulfate source (Fe_2_(SO_4_)_3_·5H_2_O) at a concentration of 0.2 M was neutralized slowly with 1 M NaOH to a constant pH of 7. It was then kept at this pH with stirring for approximately 1 h for slight aging and crystallization. At the end of the reaction period, the solution and resulting 2-line FH were separated using a pressure filter with N_2(g)_ and a 0.1 μm filter. To remove as much of the Na and SO_4_ from the surface of the 2-line FH, the resulting wet solids were placed into 1000 mL of DI water then stirred for 6 h before further separation. This procedure was repeated 4 times. The solids were then dried at room temperature, ground with a mortar and pestle then placed in the anoxic glove boxes for 2 days to ensure that most of the surface-bound oxygen was removed from the 2-line FH. All further reactions and preparation of materials were done inside the two glove boxes mentioned above.

Batch reactions in our glove boxes were conducted using a fresh ferrous sulfate source (FeSO_4_·7H_2_O) stored in our glove boxes. This was our Fe(ii)_(aq)_ catalyst to be reacted with the 2-line FH as per our previous works with other systems.^31,41,42^ Briefly, a stock solution of 10 mM Fe(ii)_(aq)_ was made by dissolving the appropriate amount of the ferrous salt into deoxygenated DI water which had a natural pH of ∼6. The solution was left overnight to be inspected in the morning to make sure no yellow color was observed in the solution that is indicative of Fe(ii) oxidation.^22^ To confirm the Fe(ii)_(aq)_ was active, the use of Fe(ii) color strips was used to ensure that the red color indicative of the Fe(ii)_(aq)_-cyano complex appeared (Sigma-Aldrich). Then, the stock solution was diluted to the second concentration used in this work, namely 0.2 mM Fe(ii)_(aq)_, and rechecked with the Fe(ii) color strips. As a final confirmation, the reacting solutions (0.2 mM and 10 mM) were measured for Fe(ii)_(aq)_ concentrations *in situ* using the ferrozine method and a UV-VIS spectrophotometer (UV-2550, Shimadzu, Japan) at a 510 nm energy. Bulk tests for selective verification of TEM results were conducted in glove box-1. These tests include placing 400 mg of 2-line FH into 40 mL of reacting solutions comprised of 10 mM or 0.2 mM Fe(ii)_(aq)_ and catalyzing for the desired reaction times (7, 24, 48, 96, and 156 h) at a natural pH of 6. No buffer was used to keep the pH constant throughout the reactions and the pH was not monitored. This is because monitoring of pH is not currently technologically possible in LC TEM experiments. As such the monitoring of pH for the bulk test would not be representative to compare with the LC TEM experiments. In this case, 400 mg of solids were used for comparison of the TEM results to ensure that we produced enough solids for bulk characterization techniques such as XRD, Raman, and ATR-FTIR.

### 2.2 *In situ* liquid experiment

In the case of the tests done with 2-line FH at pH 8 and 10 mM Fe(ii)_(aq)_, these were done as in previous works in a static LC.^39^ The pH of the solution was adjusted to 8 *via* the addition of deoxygenated lime water before combining it with the 2-line FH solid. After which no pH buffer and no pH monitoring was done as it's not possible *via* LC TEM. For the tests done at a natural pH ∼ 6 with 0.2 and 10 mM Fe(ii)_(aq),_ the 2-line FH nanoparticles were dispersed in ethanol and drop-casted onto TEM Si_3_N_4_ liquid chips (window size: 0.03 × 0.25 mm; frame size: 2.6 mm × 2.6 mm × 200 μm, Norcada Inc.). It is worth noting that for these tests, no pH buffer was used and monitoring of pH for the *in situ* LC TEM experiments is not possible. The two pH values of 6 and 8 were selected as they are commonly encountered in natural or anthropogenic aqueous systems were the catalytic transformation of 2-line FH by Fe(ii)_(aq)_ occurs.^4,6,15,16^ During the *in situ* experiment, Fe(ii)_(aq)_ catalyst solution was pumped into the liquid cell at a flow rate of 2 mL min^−1^ for TEM observation at different time durations. The preparation of Fe(ii)_(aq)_ solutions, loading of Fe(ii)_(aq)_ solutions into the syringe and plastic tubes, sealing of tube end with Fe(ii)_(aq)_ solution were done in glovebox-2 under N_2(g)_ environment. The only exception was for the assembly of the liquid cell chips which only involves Fe(iii) [as 2-line FH] and not any Fe(ii)_(aq)_ that could be oxidized. The solution loaded syringe and sealed plastic tubes were taken from glovebox-2 and connected to the liquid TEM holder. Then the liquid inside was pumped at a rate of 5 mL min^−1^ to fill the liquid channel and cell in the TEM holder. To avoid liquid leaking in the TEM from the breakage of the liquid cell chips, we tested the holder in a vacuum chamber for 5–10 min with continuous flow of Fe(ii)_(aq)_ solution. After the leak tests, we put the liquid TEM holder assembly into our TEM for *in situ* experiments while maintaining the flowing of the Fe(ii)_(aq)_ solution using a pump rate as noted above.

### 2.3 Semi *in situ* liquid experiment

The 2-line FH dispersed in ethanol was drop-casted onto TEM carbon grids (300 mesh, TED PELLA, INC.), and their relative initial locations were recorded for further inspection after the desired reaction times in 0.2 mM and 10 mM Fe(ii)_(aq)_ at a pH ∼ 6. No pH buffer was used nor pH monitored due to similar reasons aforementioned. Rather a pH drift mode was employed because accurate pH measuring of the reaction at these scales (μL and nano–micro grams) within the space of the carbon grids is not possible. Furthermore, as we observed in this work, bulk measurements may or may not be directly comparable with what we observe at the micro/nano TEM scale. It is noted that 0 h in the semi-*in situ* experiment indicates imaging before immersion in the Fe(ii)_(aq)_ solutions. All samples were prepared and loaded onto the TEM holder inside the glove box-2 filled with N_2(g)_. Furthermore, the holder with samples was protected under N_2(g)_ environment using an N_2(g)_ filled bag while transferring the TEM cells and grids from inside the glovebox to the environmental TEM to avoid the oxidation of samples.

### 2.4 Materials characterization

In the case of the *in situ* liquid cell TEM tests with 2-line FH at pH 8 and 10 mM, a JEOL 2010F microscope operating at 200 kV with a beam current of ∼35 pA cm^−2^ operating at ambient conditions using a custom made TEM holder from Hummingbird Scientific was employed as in previous works.^39^ The collection of the images in the movie† presented was done at a rate of 5 frames per second for 5 minutes.

For the tests done on 2-line FH at pH ∼ 6 with 0.2 and 10 mM Fe(ii)_(aq)_, an aberration-corrected environmental TEM (ETEM, Titan from FEI ThermoFisher Scientific, USA) was employed at 300 kV for high-resolution TEM (HRTEM) imaging, bright-field (BF) imaging and selected area electron diffraction (ED). Another aberration-corrected transmission electron microscope (Titan from FEI ThermoFisher Scientific, USA) equipped with a high-angle annular dark-field (HAADF) detector and an electron energy loss spectroscopy (EELS) system was employed at 300 kV for HAADF scanning TEM (STEM) and valence analysis. In this case, the images for the movie† presented were done at 15 frames per second for 2 minutes. The STEM-EELS data was collected in dual-EELS mode, *i.e.*, both zero-loss and core-loss spectra were collected simultaneously. Core-loss EELS were calibrated by the corresponding zero-loss EELS using DigitalMicrograph (Version 2.11, Gatan Inc.).

XRD was conducted on selected powders using a Bruker D8 advanced diffractometer with a graphite crystal, a scintillator detector as a monochromator, and Cu-K_α_*λ* = 1.5406 Å radiation. Scans were recorded from 5 to 80° 2*θ* using a 0.02° step size with a scanning speed of 2.5° min^−1^. Identification of the possible iron(iii) oxy/hydroxide phases formed along the bulk reactions (2 line-FH (PDF 98-011-1015), 6 line-FH (PDF 98-007-6750), LP (PDF 98-001-2041), GT (PDF-98-003-4786), MG (PDF-98-011-7729), HT (PDF 98-011-9589), fougerite-FG (98-011-2393)) was done on the X'pert HighScore Plus software using the PAN-Inorganic and Mineral Crystal Structure Database version 2.0.23.

Raman data on selected powders were collected using a Thermo DXR Raman Microscope from Thermo Fisher Scientific operating with a 780 nm laser using the 50× short distance objective and a laser output of 10%. Scan ranges from 150–2000 cm^−1^ were gathered with an average of 10 scans to improve data statistics. For the ATR-FTIR data collection, measurements were performed on a Nicolet 6700 Fourier Transform Infrared Spectrometer using a diamond ATR cell from Pike Technologies. The scan ranged used was 600–4000 cm^−1^ and 64 scans were co-added with a spectral resolution of 4 cm^−1^.

## 3. Results and discussion

### 3.1 Formation of precursor nano-particles from Fe(ii)_(aq)_ solutions in the absence of 2-line FH

In our work, *via in situ* LC TEM, we flowed a 10 mM Fe(ii)_(aq)_ solution without the presence of 2-line FH at pH ∼ 6 for a duration of 6, 17, and 30 h (Fig. S1†) and noticed that some nano-particles formed as well as deposited on the Si_3_N_4_ film. At this point, it is worth noting that significant precautions to avoid any possible oxidation from the air was undertaken as outlined in the Experimental section. Unfortunately, due to the lack of good resolution to image these nano-particles precipitating from the Fe(ii)_(aq)_ solutions *in situ*, we further decided to investigate their formation and identity *via* semi *in situ* tests. Immersion of clean carbon grids in 10 mM Fe(ii)_(aq)_ solutions and inspection of them after 3 h again showed that some amorphous like nano-particles precipitated (Fig. S2†). Exposure to a longer time of 24 h (Fig. S2†) further revealed that some semi-crystalline nano-particles formed. Electron diffraction (ED) indicated them to likely match with GT and/or HT. However, the formation of HT is not likely to precipitate under our reaction conditions as HT tends to usually precipitate at higher pHs and temperatures.^2,43–46^ To further verify their identity, an extension of 48 h (Fig. S2†) revealed the aggregated nano-particles were elongated rod-shaped and their ED indicated them to be polycrystalline GT. A final extension of time to 72 and 156 h (Fig. S2†) further confirmed our previous results as only further crystallization and growth were observed.

Decreasing solution concentrations to 0.2 mM Fe(ii)_(aq)_ at a pH ∼ 6 after 48 h revealed the formation of agglomerated nano-particles (Fig. S3†) similar to those observed at higher concentrations and equal times. Inspection of their ED patterns revealed them to consist of polycrystalline LP. A closer inspection revealed them to be composed of agglomerated round particles as well as nano-wires/needles (Fig. S3†) with ≤5 nm widths and various lengths. Further extension of time to 96 h revealed agglomeration of these nano-particles similar to what was observed at 10 mM and comparable reaction times (Fig. S3†). ED on particular regions (Fig. S3†) indicated them to consist of a polycrystalline LP and some amorphous component.

Bulk based analysis of 0.2 mM and 10 mM Fe(ii)_(aq)_ solutions at a natural pH ∼ 6 were protected from oxidation by placing them in sealed capillaries inside our glovebox-1 and analyzed *via* Raman at various times. The data (Fig. S4†) only showed the presence of water and the Fe(ii)SO_4_ solution inside the capillary. Similarly, rapid (<1 min in the air) ATR-FTIR analysis of these solutions at both concentrations showed only the presence of water and SO_4_ groups (Fig. S5†). Neither vibrational techniques showed indications of GT, LP, 2-line FH, or any other possible solid Fe(ii), Fe(ii)/Fe(iii), and/or Fe(iii) oxy/hydroxide. The lack of detection of these particles observed in our *in situ* liquid cell and semi *in situ* TEM analysis likely arises from the fact that their concentration is far too low to be detected by these bulk measurements. Further thermodynamic calculations conducted at 10 mM and pH 6 revealed that both amorphous and crystalline Fe(OH)_2(s)_ were under saturated and as such no precipitation was predicted to occur (Fig. S6†).

The presence of these nanoparticles from only Fe(ii)_(aq)_ solutions under our experimental conditions was rather unexpected as they have never been reported in the literature.^20–23,26–30^ Fe(ii)_(aq)_ solutions were carefully protected from oxygen oxidation to avoid precipitation of Fe(iii) oxy/hydroxides. As such we do not believe the nano-particles observed were a product of oxidation from O_2(g)_ and/or air. Furthermore, Fe(ii)_(aq)_ quantitative measurements *via* the ferrozine method using our concentrations (*i.e.* 0.2 mM and 10 mM) and reaction times through a control experiment have demonstrated that its oxidation is negligible (Fig. S7†). This is in agreement with recent work by Sheng *et al.*^29^ who also indicated that Fe(ii)_(aq)_ oxidation is negligible at similar concentrations and reaction times without the presence of 2-line FH. Some recent reports^47,48^ have indicated that high energy electrons generated by the TEM in liquids can create hydrated electrons (e_H_), and a rich amount of radicals species such as H˙ and OH˙. The oxidation of Fe(ii) *via* radical redox reactions with OH˙ and/or SO_4_˙^−^ has been previously documented^49^ and as such the possibility of this occurring in our system is feasible. Such radical reactions would explain the formation of Fe(iii) oxy/hydroxide phases observed from only Fe(ii)_(aq)_ solutions that were not subject to air and/or O_2(g)_ oxidation. As such, the modified solution chemistry of the samples under the e-beam may induce spontaneous precipitation of nano-particles within seconds to minutes of exposure. Thus the possibility of the nano-particles observed in our study arising from radical oxidation–reduction reactions from a dose rate of ∼1.0 × 10^4^ e/nm^2^ s and/or confinement effects is conceivable. The specific effects of varying the e-beam dosage on these reactions are unclear at this point but will be investigated in more detail for continuing future work.

Therefore, the formation of these nano-particles from only Fe(ii)_(aq)_ at early stages highlights precursor phases that occur in these reactions such as those that may occur during the photo-oxidation of Fe(ii)_(aq)_ in natural environments where band iron formation occurs.^50^ However, they have not yet been observed because these types of reactions have never been monitored *via* LC TEM. Furthermore, such precursor nano-particles can't be observed with conventional bulk lab or synchrotron characterization methods because they are too low in concentration and dispersed in the liquid to detect. Further work in progress not presented here on other systems (*e.g.* boehmite and LP) *via* LC-TEM have also shown these types of precursor nano-particles to form from Fe(ii)_(aq)_ solutions without the presence of any reacting solids.

### 3.2 Dissolution of 2-line FH

The reductive dissolution of 2-line FH by Fe(ii)_(aq)_ has been indirectly inferred from various analytical methods of the final reaction products.^13,20,21,28,29^ This inference has been done based on the fact that traces of the 2-line FH signals are detected at the end of the reaction period.

**Fig. 1:**
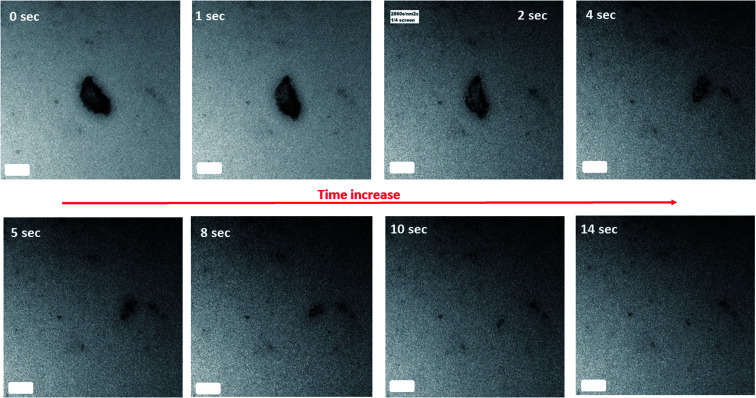
*In situ* LC-TEM measurement of the dissolution of 2-line FH in 10 mM Fe(ii)_(aq)_ at pH ∼ 6 with reaction time. The scale bar on all images is 100 nm. Refer to Movie 1† for real-time dissolution events.

In our work, using *in situ* LC TEM, we visually observed (Fig. 1 and Movie 1†) the direct dissolution of the 2 line FH particles overtime at pH ∼ 6 and 10 mM Fe(ii)_(aq)_. Our analysis was conducted with or without e^−^ beam exposure and in the presence or absence of Fe(ii)_(aq)_ solutions (Fig. S8†). This was done to ensure that the observed dissolution was a direct cause of the reduction reaction of the 2-line FH with the Fe(ii)_(aq)_ ions and not solely from possible e^−^ beam effects. However, it should be noted that studies by Pan *et al.*^51^ have indicated only when doses were >3 × 10^8^ e per nm^2^ was the crystal structure and electronic structure of FH altered. At e-doses such as the ones we used in our work of ∼1 × 10^4^ e per nm^2^, no major phase or electronic structural changes are expected to be observed. To further verify this dissolution process, semi *in situ* analysis of tracked 2-line FH particles in 10 mM and 0.2 mM by Fe(ii)_(aq)_ solutions at various times was also performed (Fig. S9†). In general, we observed that particles that were reacted at high concentrations of 10 mM Fe(ii)_(aq)_ (Fig. S9†) decreased in size and became less dense (from the contrast of the images) as they dissolved with time. This is similar to observations made by Li *et al.*^52^ on the dissolution of HT nano-particles. In our work, however, the 2-line FH particles were much larger and we did not yet systematically study the effects that crystal defects may have had on the dissolution. However, based on previous works,^52^ likely some influence of crystal defects on its dissolution may occur. Lowering the concentration to 0.2 mM Fe(ii)_(aq)_ (Fig. S9†) showed a similar behaviour but the effect was less pronounced over the studied reaction time. Therefore, even though this concept of dissolution of 2-line FH by Fe(ii)_(aq)_ has been proposed for decades, this is the first time it has ever been physically observed *in situ*. Moreover, as highlighted here, the reductive dissolution appears to go through a process where the particles decrease in size and density.

Furthermore, we further observed that the dissolution of the 2-line FH particles had not always gone to completion with 0.2 mM or 10 mM Fe(ii)_(aq)_ as the reaction time varied from 6 h up to 96 h (Fig. S10†). This was observed to occur for both large (≥1 μm) and smaller (≤1 μm) size particles. Hence indicating that at these micro/nano scales, the apparent particle size may not have such an influence on the dissolution of 2-line FH as observed for HT.^52^ This is in agreement with previous studies^13,20,21,53,54^ where a lack of transformation (“induction period”) on the reductive dissolution of 2-line FH by Fe(ii)_(aq)_ has been indirectly inferred *via* spectroscopic and diffraction analysis. However, such analytical techniques give an average ensemble of all particles reacted within the sample and as such lacks the spatial specificity that our semi *in situ* TEM analysis offers. The exact reasons for such an “induction period” as observed here are unknown but may be a result of some type of surface Fe(ii) passivation that occurs on the surface of the 2-line FH particles as recently noted for GT.^55^ Moreover, the differences in dissolution times observed (Fig. 1-short *vs.* Fig. S10-long†) may be due to the surface Fe(ii) passivation content varying from particle to particle at the micro/nano-scale and/or possible Fe(ii)_(aq)_ gradients that may not reach all particles equally. Thus, our work gives the first visual confirmation that an “induction period” occurs when 2-line FH reacts with relatively high and low Fe(ii)_(aq)_ concentrations, it can last for a relatively extended time (up to 4 days) and it appears to be independent of the size of the particles.

### 3.3 Amorphous phase growth and deposition on the surface of 2-line FH

It has been noted in the existing literature that upon the adsorption of Fe(ii)_(aq)_ onto the surface of 2-line FH, oxidation of the adsorbed Fe(ii) induces the growth of a Fe(iii) layer. This Fe(iii) layer is similar to the bulk 2-line FH and has recently been labeled as a “reactive-FH/labile Fe(iii) phase”.^20,22,29,30,56^ The presence of this species has been inferred from spectroscopic and diffraction signals. Also, this inference has been suggested from an increase in the size of the 2-line FH particles observed *via ex situ* TEM after exposure to Fe(ii)_(aq)_ solutions for 2, 3, and 6 hours.

**Fig. 2:**
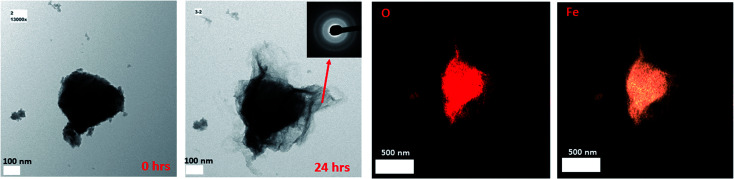
The reacted 2-line FH in 10 mM Fe(ii)_(aq)_ at pH ∼ 6 showing the amorphous layer on the surface of the reacted particle as determined by ED and its corresponding chemical composition *via* EDX.

**Fig. 3:**
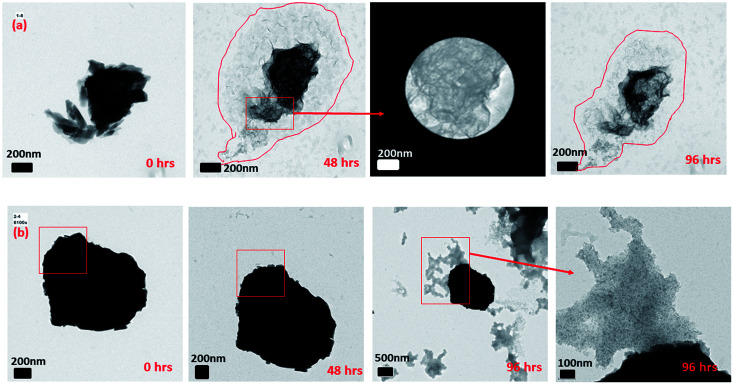
(a) Shows a diffuse cloud (“aura”) of nano-particles around the reacted 2-line FH with 0.2 mM Fe(ii)_(aq)_ at pH ∼ 6 after 48 and 96 hours. Also displayed is a selected diffuse region of the reacted particle after 48 hours. (b) Illustrates the deposition/attachment of an amorphous phase to the 2-line FH when it is reacted with 10 mM Fe(ii)_(aq)_ at pH ∼ 6.

In our work, we also observed the growth of the 2-line FH particles when exposed to 10 mM Fe(ii)_(aq)_ and identified this amorphous surface layer that may be the so-called (“reactive-FH/labile Fe(iii) phase”)^20,29,30^ to be composed of a similar chemical composition as the bulk 2-line FH (Fig. 2 and S11†) using EDX. Furthermore, ED on the outer surface regions of the 2-line FH reacted particles show it to be composed of an amorphous component similar to 2-line FH (Fig. 2 and S11†). Lowering the reacting concentration to 0.2 mM Fe(ii)_(aq)_, also demonstrated an amorphous type of component on the surface of the 2-line FH. Although at this lower concentration, we curiously noted a diffuse cloud (“aura”) around the reacted 2-line FH (Fig. 3a) that will be further discussed in the following section. It is worth noting that in our work, the reaction times monitored (*i.e.* 24, 48, and 96 h) were much longer than previous works.^20,22,29,30,56^ However, we still observed this amorphous phase (so-called “reactive-FH/labile Fe(iii) phase”)^20,29,30^ on the surfaces of various reacted 2-line FH particles at the micro/nano-scale. This further confirms our observations from Section 3.2 that indeed there is a significant “induction period” on the transformation of 2-line FH by Fe(ii)_(aq)_ at both high and low concentrations even though we may associate higher Fe(ii)_(aq)_ concentrations (*e.g.* 10 mM) with higher solid associated Fe(ii)-2-line FH and thus faster conversion at the bulk scale.^20^ Therefore, this work for the first time visually reports the chemical and phase composition of the amorphous surface phase that may be the so-called (“reactive-FH/labile Fe(iii) phase”)^20,29,30^ that forms when Fe(ii)_(aq)_ adsorbs to 2-line FH and induces a surface growth of a Fe-layer.

Herein, we also observed the direct deposition of amorphous aggregated nano-particles on the surface of the 2-line FH particles (Fig. 3b and S12†) that were not dissolved over our reaction times (*i.e.* 24, 48, and 96 h). We may infer that these aggregated nano-particles that attach on the surface of the 2-line FH may be the precursor nano-particles we noted in Section 3.1 and may be similar to the colloidal precursors observed by Banfield *et al.*^57^ Therefore, this type of observed attachment may be a continuous process that may cause growth and/or oriented attachment during the catalytic transformation of 2-line FH by Fe(ii)_(aq)_ but has yet to be reported until now. Future work *via in situ* liquid cell TEM to provide real-time imaging of these events will be conducted to further investigate the role these aggregated attached nano-particles play in the catalytic transformation of 2-line FH by Fe(ii)_(aq)_.

EELS analysis of the 2-line FH particles before and after immersion in 10 mM Fe(ii)_(aq)_ for 34 h (Fig. S13†) done in semi *in situ* mode revealed the presence of Fe(ii)/Fe(iii) at the surface of the outer edges of the reacted 2-line FH particles. Therefore, this observation visually demonstrates for the first time that the amorphous phase developed and/or the deposited aggregated nano-particles on the surfaces are indeed composed of an Fe(ii)/Fe(iii) interface as previously proposed by Tronc *et al.*^58^ and Jolivet *et al.*^59^ but never yet physically observed. The presence of this Fe(ii)/Fe(iii) at the surface of the reacted 2-line FH particles after 34 h also highlights the fact that the Fe(ii) adsorbed lifetime extends what we would normally expect.^20,29,30^ As such, the formation and lifetime of the amorphous “reactive-FH/labile Fe(iii)”^20,29,30^ phase which arises from the oxidation of the adsorbed Fe(ii) on the reacted 2-line FH at the nano/micro-scale may be different then we presume. Moreover, the presence of the Fe(ii)/Fe(iii) at the surface of the reacted 2-line FH particles after such a long period of time may contribute to the “induction period” as observed in Section 3.2 where a possible passivation Fe(ii) layer may form on 2-line FH that prevents further electron transfer and phase growth/transformation as noted for GT.^55^ Finally, the presence of the Fe(ii)/Fe(iii) at the surface of the reacted 2-line FH particles verifies the adsorption of Fe(ii)_(aq)_ onto the reacted 2-line FH occurs and will eventually promote the growth of surface phases (*e.g.* “reactive-FH/labile Fe(iii)”, LP, GT) on 2-line FH as previously proposed.^20,21,29–31^

### 3.4 Release of nano-particles/wires from 2-line FH and assemblage of lamellae to form LP

**Fig. 4:**
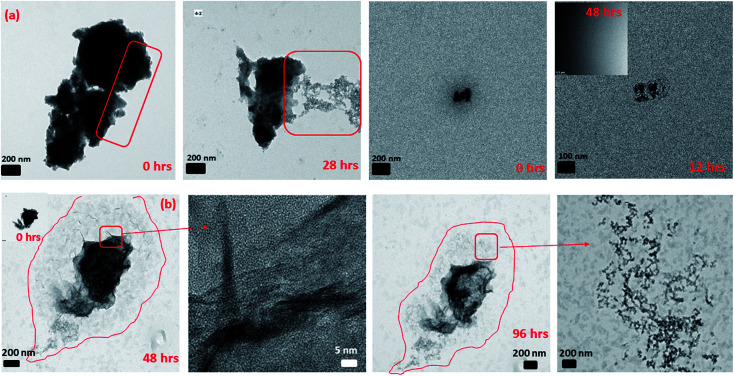
Semi *in situ* measurement of the dissolution of 2-line FH in (a) 10 mM Fe(ii)_(aq)_ at pH ∼ 6 and various reaction times. The images display the dissolution process occurring through the breakage of the particles into smaller nano-particles through pores and hollow channels. (b) 0.2 mM Fe(ii)_(aq)_ at pH ∼ 6 and various reaction times. The images display the dissolution process occurring through the breakage of the particles into smaller nano-particles as well as nano-wires and nano-whiskers coming from the surface of the reacted particles and been released.

In Section 3.2, we observed that 2-line FH dissolved completely or to some extent with time by decreasing in size and density. However, from further data collected, we also observed that at 10 mM Fe(ii)_(aq)_ and pH ∼ 6 (Fig. 4 and S14†), another type of dissolution mechanism occurred. The dissolution process also proceeded by breaking down into smaller nano-particles through pores and hollow channels similar to what was observed for HT.^52^ A closer inspection of these broken-down nano-particles revealed them to be composed of an amorphous component as well as nanowires (Fig. 4 and S14†) whose ED analysis indicated them to likely exists as GT. Lowering concentrations to 0.2 mM Fe(ii)_(aq)_ and pH ∼ 6, we observed a similar behaviour but was even more prominent in the release of the nano-particles and nano-wires as the reaction proceeded (Fig. 4 and S15†). However, in this case, the nanowires also appeared as whiskers coming out from the surface of the 2-line FH particles. Hence, the deposition of Fe(ii)_(aq)_ onto the surface of 2-line FH causes an Fe(ii)/Fe(iii) interface to form (Section 3.3) that results in the reduction–oxidation, nucleation and growth of the surface nanowires/whiskers observed here. In general, such dissolution behaviour and observation of surface nano-wire particles have not been previously reported.^20,21,29^ However, this other type of dissolution process and surface formation of nano-wires may give us a glimpse into the starting bulk formation of LP and GT *via* nano-wires to lamellae self-assembly as will be called to attention below. Future work to observe *in situ* the exact pathways for which the 2-line FH particles breakdown into the amorphous, and nano-wires as well as form the surface nano-wires/whiskers will be conducted.

**Fig. 5:**
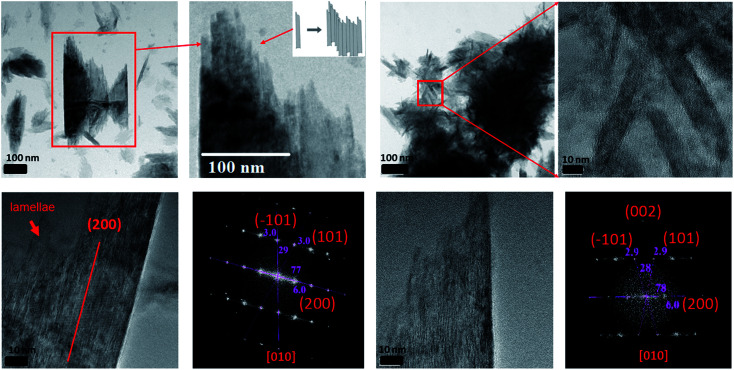
The formation of lepidocrocite tabular-like crystals from small lamellae of elongated crystals after the reaction of 2-line FH with 10 mM Fe(ii)_(aq)_ at pH ∼ 6 for 6 hours.

Previous studies have reported that LP crystals formed from the catalytic transformation of 2-line FH by Fe(ii)_(aq)_ consist of tabular plate-like crystals.^13,21,28,29,60^ Unfortunately, these reported LP particles have been well-crystallized and imaged after long reaction times (*e.g.* 5.5, 9, and 30 days). In our work, after 6 h of reaction with 10 mM Fe(ii)_(aq)_, we observed the formation of small lamellae of elongated crystals which were found to be oriented in various directions (Fig. 5 and S16†). Furthermore, we imaged large tabular plate-like crystals that were composed of and assembled from these elongated crystals (Fig. 5 and S16†). Hence from these images, we observed a glance of how the assemblage of these tabular LP crystals to form the larger plate-like crystals occurs. Furthermore, we also observed how singular tabular ones that had not yet assembled into larger particles formed. ED of the larger elongated crystals revealed them to be LP (Fig. 5 and S16†). Therefore, we may infer that the nano-wires and amorphous particles that are released from the dissolution of 2-line FH as noted above and perhaps even the precursor nano-particles from on Fe(ii)_(aq)_ solutions in Section 3.1 may eventually form these elongated crystals that are then assembled into larger tabular LP particles. Future work, to observe these events *via in situ* liquid cell TEM will be conducted to further solidify these findings but thus far, our semi *in situ* data presented here support these ideas.

### 3.5 Goethite and lepidocrocite end products

Bulk batch reactions at 10 mM and pH 6 were conducted after 7, 24, 48, 156 h and analyzed *via* XRD, Raman, and ATR-IR (Fig. S17 and S18†). The bulk analysis revealed that GT is the major transformation phase along with small amounts of LP and some remaining 2-line FH. Semi *in situ* TEM analysis (Fig. S19†) of the reacted particles after 7 h showed the presence of the remaining 2-line FH, some tabular shaped crystals (LP), and elongated crystals (GT). Furthermore, we observed nano-wires dispersed as well as growing out of the surface of the reacted 2-line FH, similar to what was observed in Section 3.4. Extension of the reaction time to 24, 48, and 156 h (Fig. S19†) further confirmed the presence of elongated and star-shaped GT crystals as observed in other works.^20,21^ ED analysis showed mainly the presence of GT but also some planes attributed to LP (Fig. S19†). In our case, the presence of MG was not observed even at these high Fe(ii)_(aq)_ concentrations because the pH was not sufficiently high enough for MG precipitation to occur.^20,21,61^ Therefore, these results show that the added Fe(ii)_(aq)_ indeed had catalysed the 2-line FH particles^20–22,26–31^ as the reactions proceeded to the transformation of the expected end products.

Lowering the concentration to 0.2 mM at pH 6 for similar reaction times showed only the presence of 2-line FH *via* bulk XRD, Raman, and ATR-IR analysis (Fig. S17†). Semi *in situ* TEM analysis (Fig. S20†) similarly showed mainly the presence of 2-line FH particles but a GT like particle was identified after 48 h. More importantly, we observed at the micro/nano-scale, the growth of nano-wires coming out of the surface of the reacted 2-line FH as noted in Section 3.4. Furthermore, the presence of aggregated amorphous nano-particles that resembled those observed arising from only Fe(ii)_(aq)_ solutions (Section 3.1) was also observed. Thus indicating that although the bulk characterization techniques could not detect some of the reaction products (*e.g.* nanowire and surface nano-whiskers), these reactions with the added Fe(ii)_(aq)_ proceeded to react but changes could only be observed/detected at the micro/nano-scale.

### 3.6 Possible movement of adsorbates on the surface of a Fe(iii)-oxy/hydroxide crystal

Upon reaction of 10 mM Fe(ii)_(aq)_ with 2-line FH at pH ∼ 8 for a period of 1.5 h in the liquid cell, we began to observe that an elongated crystal had distinct movements on its surface typical of bend contours (Fig. S21, Movies 2 and 3†). Such movements could be caused by surface stresses/strains from adsorption of ions in solution onto the crystal surface and/or small motions under the e^−^ beam. Noteworthy is the fact that these bend contours are completely different then pull–push stress/strain tests reported on metal/alloys *via in situ* TEM measurements^62–64^ and that a similar kind of phenomenon but not exactly the same may be observed in the connection of calcite rhombohedra in Nielsen *et al.*^65^ (Movie 6† therein) but not yet noticed/reported until now.

From the images and TEM videos, we can infer that several distinct events occur based on the movement of the bend contours: (1) a deposition (“coating”) on the surface of the crystal, (2) redistribution of the hypothesized adsorbed particles through some kind of zoning to different regions of the crystal, (3) the repulsion and attraction of one of the zoning bands until reaching a higher concentration zone (*i.e.* darker image contrast) on the crystal, (4) a second deposition (“coating”) on the surface of the crystal, and (5) a static event whereby all the motion of the bend contours have come to a rest.

Unfortunately, due to current technological limitations that are encountered with the detection of mono or bilayer deposition of ions in solution onto crystals in a liquid cell *via* EDX and/or EELS, for now, we could not yet definitively verify *in situ* the identity of the adsorbing material on the crystal. The most obvious identity for the adsorbing adsorbate is our Fe(ii)_(aq)_ but also SO_4(aq)_ ions are a possibility because our catalyst reagent is in the form of FeSO_4_·7H_2_O. This inability to definitively identify the adsorbate primarily arises from the small amount of material deposited. Moreover, challenges associated with the differentiation of the EDX and/or EELS signal coming from the liquid phase and what has been adsorbed exists. Furthermore, the need to show that there exists more material on the surface of the crystal than elsewhere (*e.g.* liquid phase) is still a challenge.

If however, the data presented here is the first real-time *in situ* imaging of an adsorption process of aqueous ions onto the surface of the crystal solid,^66^ then this leads us to question if the adsorption of ions from solution is a continuous process as currently described by adsorption isotherm models (*e.g.* Langmuir, Freundlich, BET, *etc.*). To date, we have only been able to gather snap-shots of steady-state (“equilibrium”) processes in the time scale of the employed technique (*e.g. ex situ* TEM/SEM combined with EDX/EELS, AFM, STM, EMPA, XPS, STXM). But from this LC TEM data, we may ponder whether adsorption events from ions in solution onto crystal solids occur *via* discrete adsorption events through possible “adsorption waves”. Moreover, once the solution ions are deposited, we currently don't know whether there exist surface energies and/or surface charge gradients that may cause attractive or repulsive forces to redistribute the adsorbed ions to discrete regions of the crystals.

Presuming that the deposition (“coating”) observed in events 1 and 4 are the Fe(ii)_(aq)_ ions, this would then agree/complement our observations of Section 3.3 where the presence of Fe(ii)/Fe(iii) at the surface of the reacted 2-line FH particles were detected. Furthermore, its zoning into various regions of the crystals surfaces (events 2 and 3) may help to understand the reduction–oxidation, nucleation and preferred growth orientations of phases (*e.g.* “amorphous surface layer, nano-wires, LP, GT) observed at the surface of reacted 2-line FH particles as noted in Sections 3.3–3.5 and previous works.^8,20–22,29–31^

Future work and technological advancements in real-time *in situ* EDX/EELS LC-TEM to detect possible mono/bi-layer deposition of ions from solution onto crystals are still needed to further solidify our data presented here. However, this data is presented to stimulate discussion among the nanoscience/technology research community on the possible use of LC-TEM to observe such phenomena and existing technological bottlenecks of real-time *in situ* EDX/EELS LC-TEM analysis that prevails.

## 4. Revision of conceptual model and conclusions

Despite decades of research, the exact mechanisms by which Fe(ii)_(aq)_ catalyzes 2-line FH to more thermodynamic stable phases and the factors contributing to it are still a matter of on-going discussion.^20–23,26–31^

**Fig. 6:**
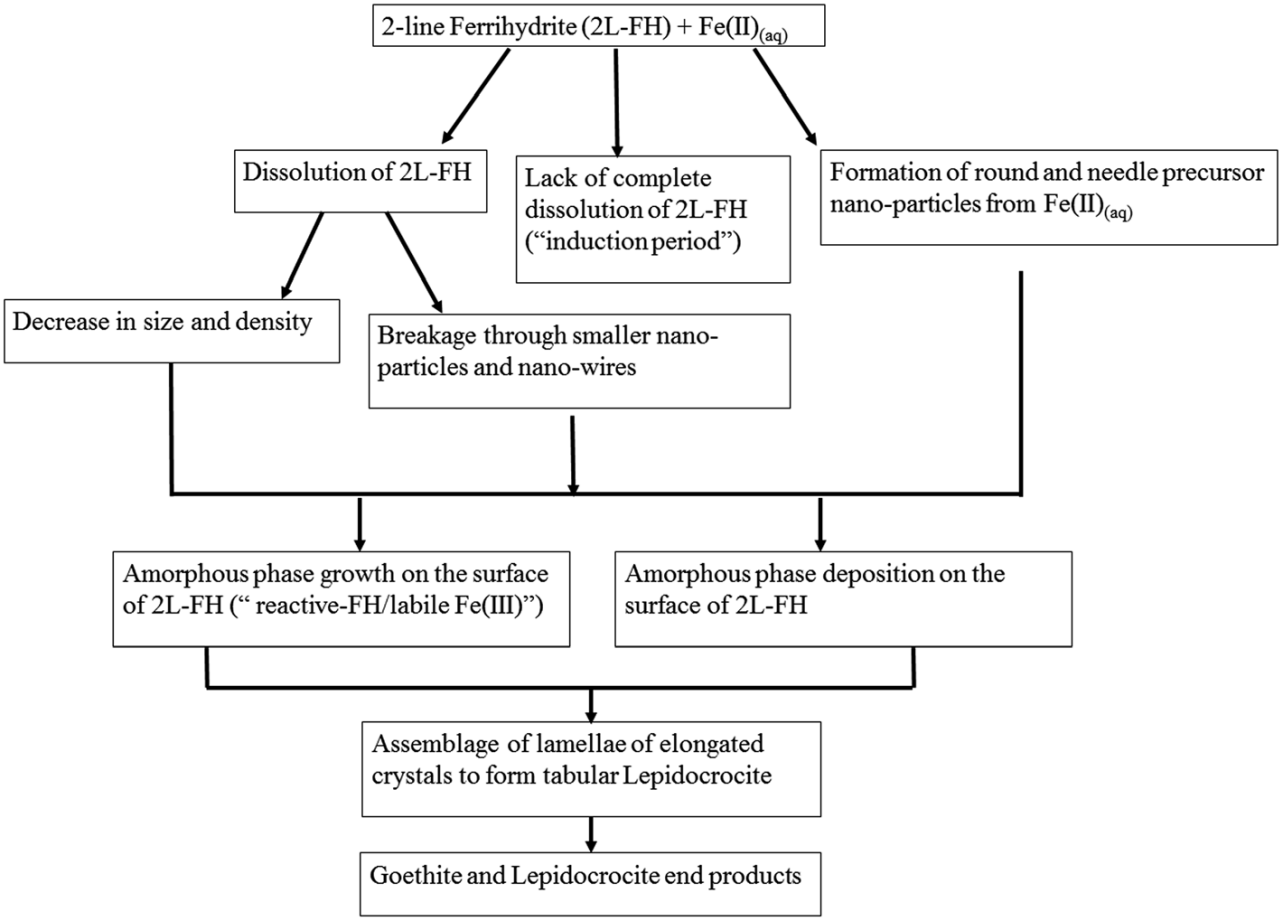
A proposed revised conceptual model for the catalytic transformation of 2-line FH by Fe(ii)_(aq)_ at a pH ∼ 6 that includes the intermediate steps observed in this work.

Herein, based on newly gathered data at the micro/nano-scale from Sections 3.1–3.6, we propose a revised conceptual model that includes these additional intermediate steps (Fig. 6). From this study, we have observed the formation of precursor nano-particles arising only from the Fe(ii)_(aq)_ solutions; something never previously reported *in situ* at the micro/nano scale.^20,21,23,29,30^ The formation of such precursor nano-particles may or may not play a role through interactions (*e.g.* attachment/deposition) with 2-line FH and/or other phases that form along the process. The lack of complete dissolution (“induction period”) and the observation of two distinct dissolution mechanisms for 2-line FH may be important factors that contribute to the environmental fate, bioavailability, and transport of Fe and as an extension any adsorbed elements (*e.g.* As, Si, P) in reducing aqueous environments. The first visual evidence of the amorphous surface layer, previously hypothesized to be a “reactive-FH/labile-Fe(iii) phase”^20,29^ observed in our work been composed of the same chemical and structural composition as the bulk 2-line FH as well as the presence of a Fe(ii)/Fe(iii) interface at the surface of the reacted 2-line FH leads us to visually solidify previous hypotheses about their possible existence.^17,20,29,58,59^ The precipitation of the well documented tabular LP crystals formed through the assemblage of elongated lamellae is a type of formation mechanism that has not yet been documented.^20–22,26–31^ Detection of LP and GT end products as well as the observation of nano-wires dispersed and growing out of the surface of the reacted 2-line FH indicates that the Fe(ii)_(aq)_ catalysed the 2-line FH particles as intended. Finally, the *in situ* LC-TEM observations from the surface bend contours give us new possible insights into the behaviour of how adsorbate species may behave once they are deposited onto crystal surfaces as well as how they may contribute to the reduction–oxidation, nucleation and growth of phases at the solid–liquid interface of minerals.

Thus our work here, using *in situ* and semi *in situ* TEM analysis provide further confirmation, new understanding, and knowledge into the mineralization mechanisms for the catalytic transformation induced by Fe(ii)_(aq)_ on 2-line FH at micro/nano-scale. Such information has not yet been observed for such systems because such techniques and methods have never yet been applied until now.

However, future work *via in situ* LC-TEM to observe in real-time some of these new intermediate events (*e.g.* attachment of nano-particles to FH, dissolution of FH, nano-wire surface growth, the assemblage of lamellae to form LP) will be undertaken. Furthermore, technological advancements to observe *in situ* EDX/EELS real-time detection of adsorbed ions deposited on solid crystal surfaces and the monitoring of their behaviour once adsorbed are still needed to be developed but would be of great interest to the research community in numerous research fields. Such applications will allow us to gain new knowledge and perspective on the chemical, structural and mechanistic sorption behaviours of sorbates onto crystal surfaces in many different material systems (*e.g.* Fe(ii)_(aq)_-2-line FH) that we have not been able to resolve by other conventional techniques.

## Conflicts of interest

We have no conflicts of interest to declare.

## Supplementary Material

NA-002-D0NA00643B-s001

NA-002-D0NA00643B-s002

NA-002-D0NA00643B-s003

NA-002-D0NA00643B-s004
