# Supplementary material for: Further insights into the Fe(ii) reduction of 2-line ferrihydrite: a semi in situ and in situ TEM study
Source: Nanoscale Adv. 2020 Sep 30;2(10):4938–50. doi: 10.1039/d0na00643b (PMC9417501; doi:10.1039/d0na00643b)
Supplement: NA-002-D0NA00643B-s004 [file NA-002-D0NA00643B-s004.pdf]

Electronic Supplementary Material (ESI) for Nanoscale Advances.
This journal is © The Royal Society of Chemistry 2020

Supplementary Information

Further insights into the Fe(II) reduction of 2-line Ferrihydrite: A semi *in-situ* and *in-situ* TEM study

Mario Alberto Gomez[1,2§], Ruonan Jiang[1], Miao Song[3§], Dongsheng Li[3], Alan Scott Lea[4], Ma Xu[5], Haibo Wang[1], Xuiling Ying[5], Shaofeng Wang[5], and Yongfeng Jia[5]

[1]*Liaoning Engineering Research Center for Treatment and Recycling of Industrially Discharged Heavy Metals, Shenyang University of Chemical Technology, Shenyang, Liaoning, China, 110142*

[2]*Department of Geological Sciences, University of Saskatchewan, Saskatoon, Saskatchewan, Canada, S7N 5E2*

[3]*Physical and Computational Science Directorate, Pacific Northwest National Laboratory, Richland, Washington, USA, 99352*

[4]*Environmental and Biological Sciences Directorate, Pacific Northwest National Laboratory, Richland, Washington, USA, 99352*

[5]*Key Laboratory of Pollution Ecology and Environmental Engineering, Institute of Applied Ecology, Chinese Academy of Sciences, Shenyang, Liaoning, China, 110016*

[§] Both authors contributed equally to this work

*Corresponding authors: Telephone:+86 15140014967；Fax: +86 15093716277

Telephone:1 5093716277；Fax: 1 5093716242

E-mail addresses: mario.gomez@syuct.edu.cn (M. A. Gomez) and Dongsheng Li2@pnnl.gov (D. Li)

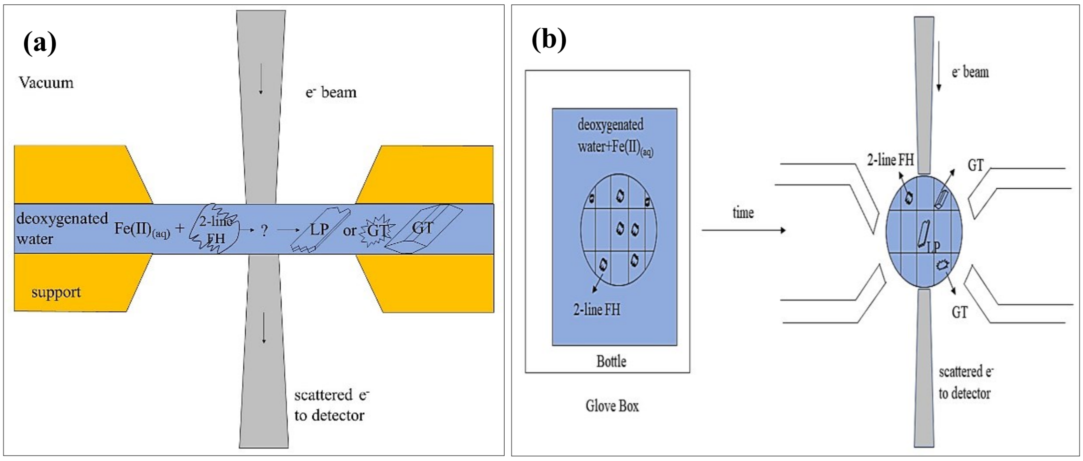

Fig. S0. Schematic of (a) *In-situ* LC-TEM and (b) semi *in-situ* TEM measurements conducted in this work to investigate at the micro/nano-scale the reaction products and possible intermediate events that occur when $Fe(II)_{(aq)}$ catalyzes 2-line FH.

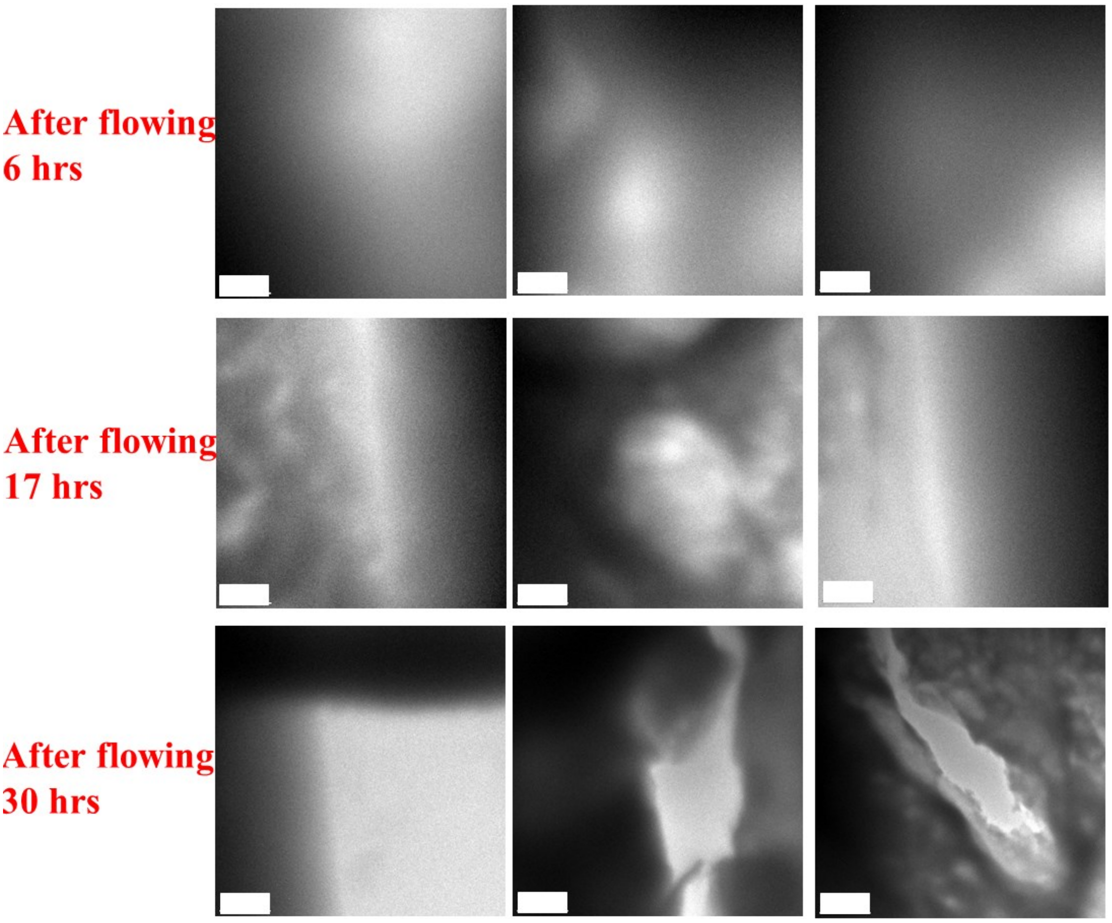

Fig. S1. Flowing of a 10mM $Fe(II)_{(aq)}$ solution in a liquid cell at a natural pH ~6 showing the formation of precipitates depositing on the Si_3N_4 film. The scale bar in all images

is 1μm. During the first 6 h, the space was filled by the $Fe(II)_{(aq)}$ solution. As time proceeded to 17 h we began to see the precipitation of some particles. Finally, after 30h of flowing the $Fe(II)_{(aq)}$ solution, many particles were found to be deposited on the Si_3N_4 film.

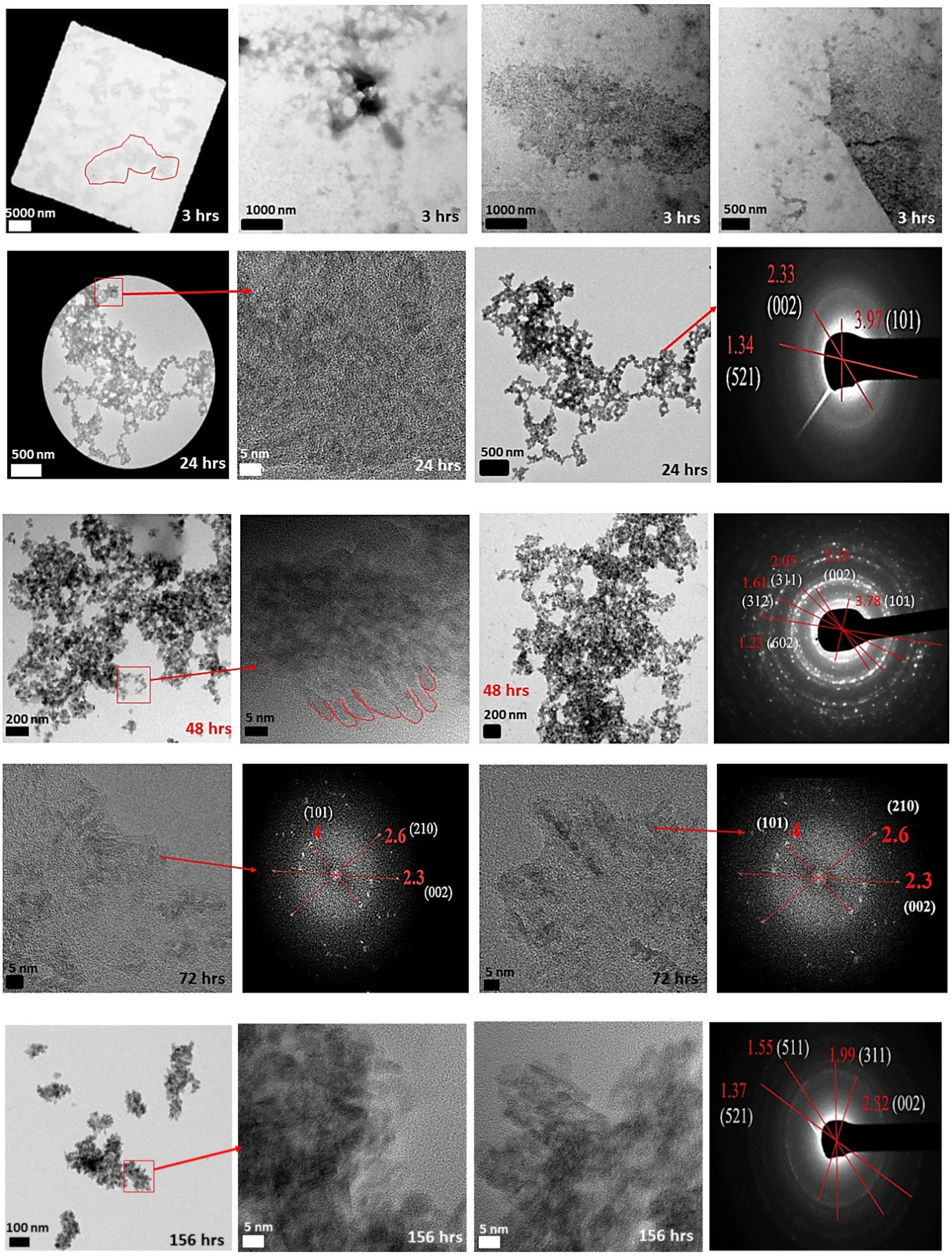

Fig. S2. Nano-particles formed at 10mM $Fe(II)_{(aq)}$ without the presence of 2-line FH at various reaction times of 3hrs, 24 hrs, 48 hrs, 72 hrs and 156 hrs. The ED patterns of

the corresponding 24, 48, 72 and 156 hrs show them likely to be in the form of Goethite, respectively.

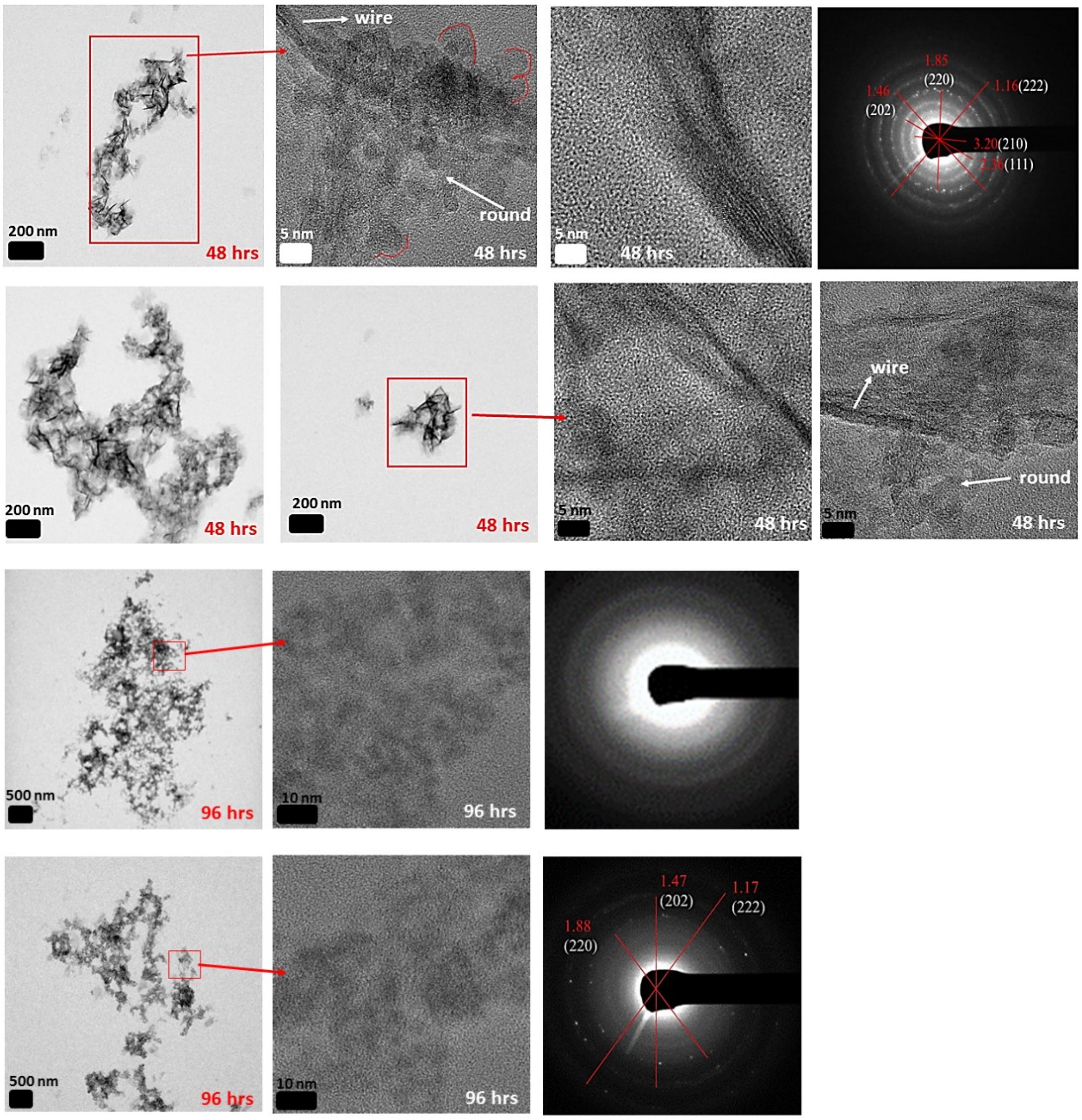

Fig. S3. Nano-particles formed at 0.2mM $Fe(II)_{(aq)}$ without the presence of 2-line FH at reaction times of 48 hrs and 96 hrs. The ED images of time 48, and 96 hrs show an amorphous component and a polycrystalline Lepidocrocite phase, respectively.

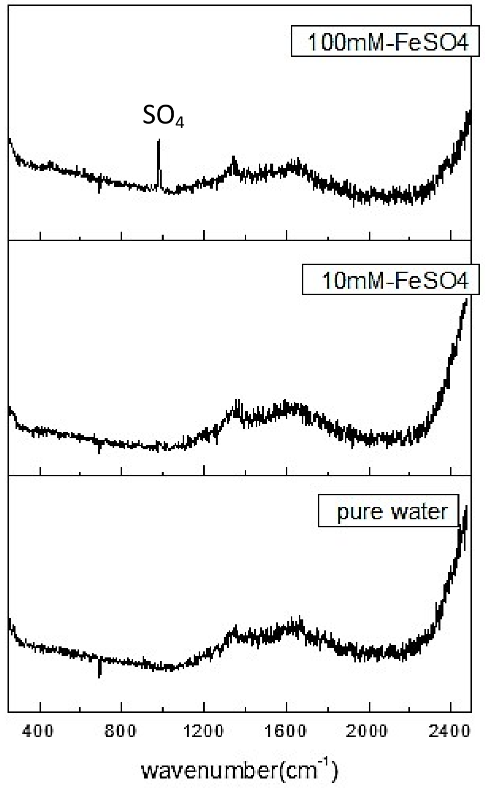

Fig. S4. Solution based inert Raman spectra of pure water, and Fe(II)SO_4 solutions at 10 and 100mM.

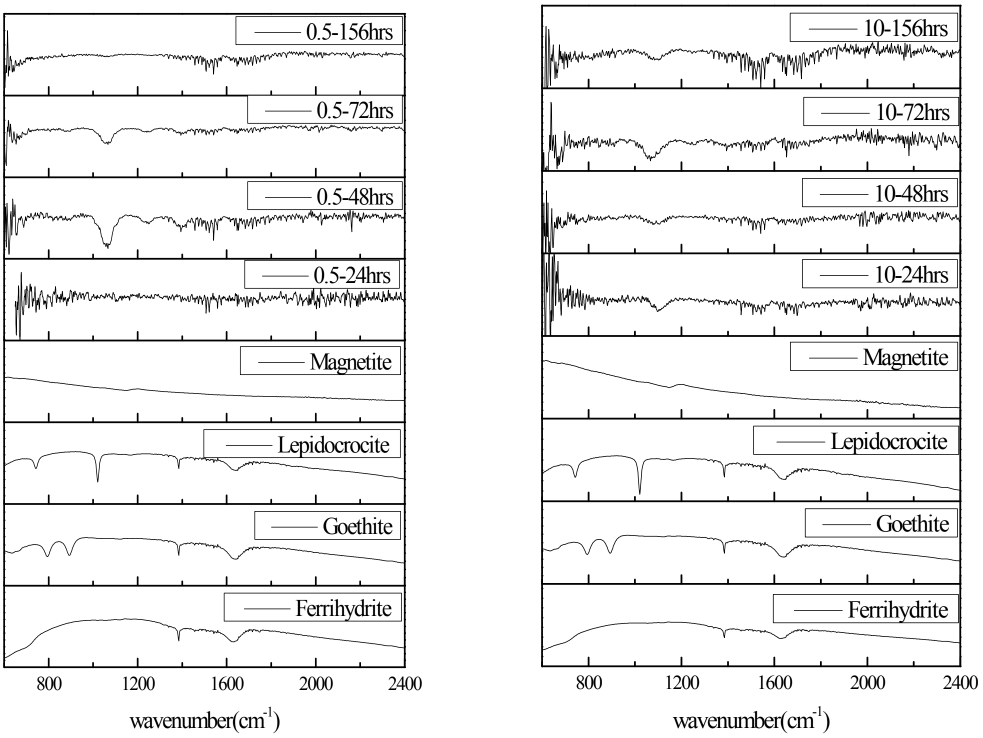

Fig. S5. Rapid solution based ATR-IR of $Fe(II)SO_4$ solutions at 0.5 and 10mM. Corresponding solid possible phases are also shown below for comparison.

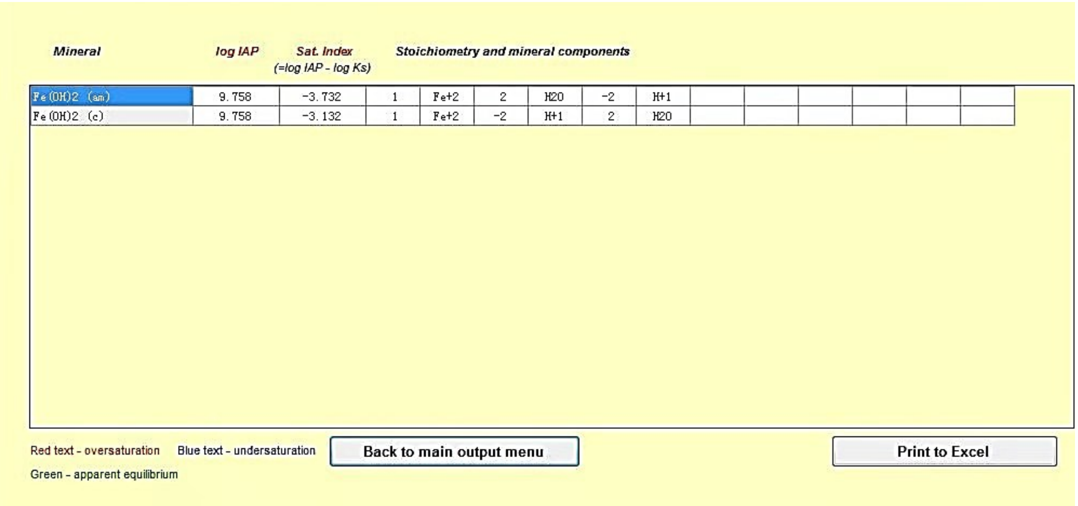

Mineral	log IAP	Sat. Index (=log IAP - log Ks)	Stoichiometry and mineral components							
Fe(OH)2 (am)	9.758	-3.732	1	Fe+2	2	H2O	-2	H+1		
Fe(OH)2 (c)	9.758	-3.132	1	Fe+2	-2	H+1	2	H2O		

Fig. S6. Saturation indexes of possible precipitation of $Fe(OH)_2$ solids at a pH of 6 and 10mM with Visual MINTEQ.

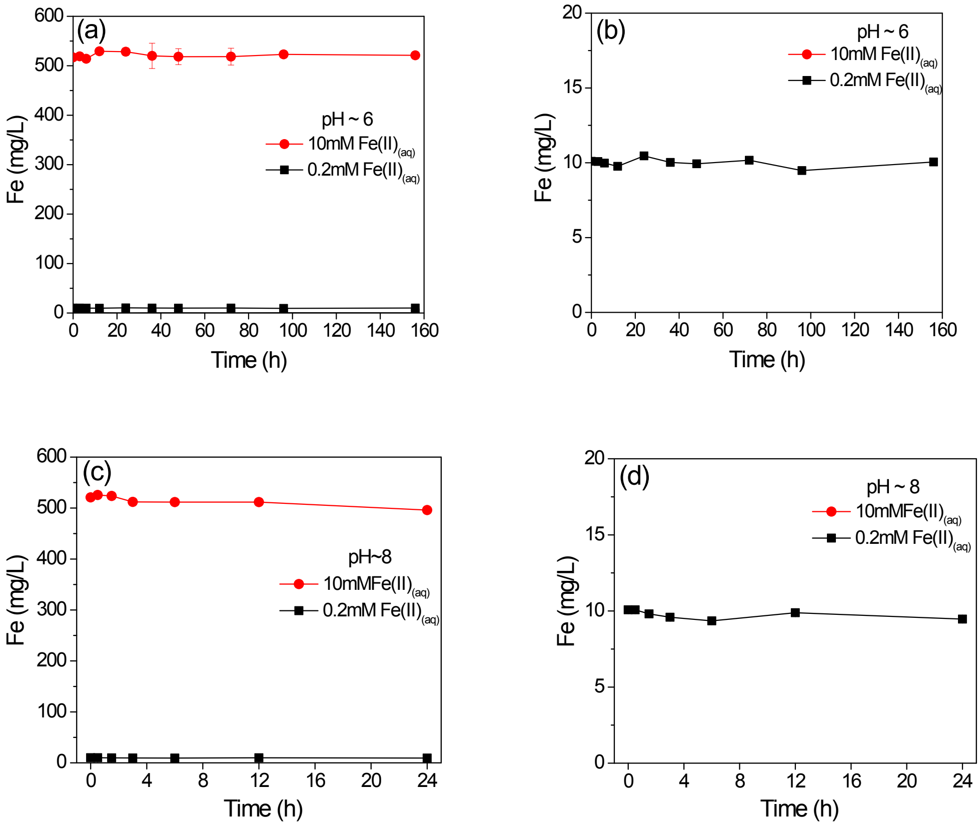

Fig. S7. $Fe(II)_{(aq)}$ evolution with time for 0.2mM and 10mM concentrations without the presence of 2-line FH at pH ~ 6 [a-b] and pH ~8 [c-d]. All tests were done in duplicates and the mean values are presented. As it can be seen from this figure, there is no significant changes in $Fe(II)_{(aq)}$ throughout the reaction periods. Thus indicating that any possible oxidation of $Fe(II)_{(aq)}$ is negligible under our reaction conditions and in agreement with Sheng et al.[29] who also observed a similar trend.

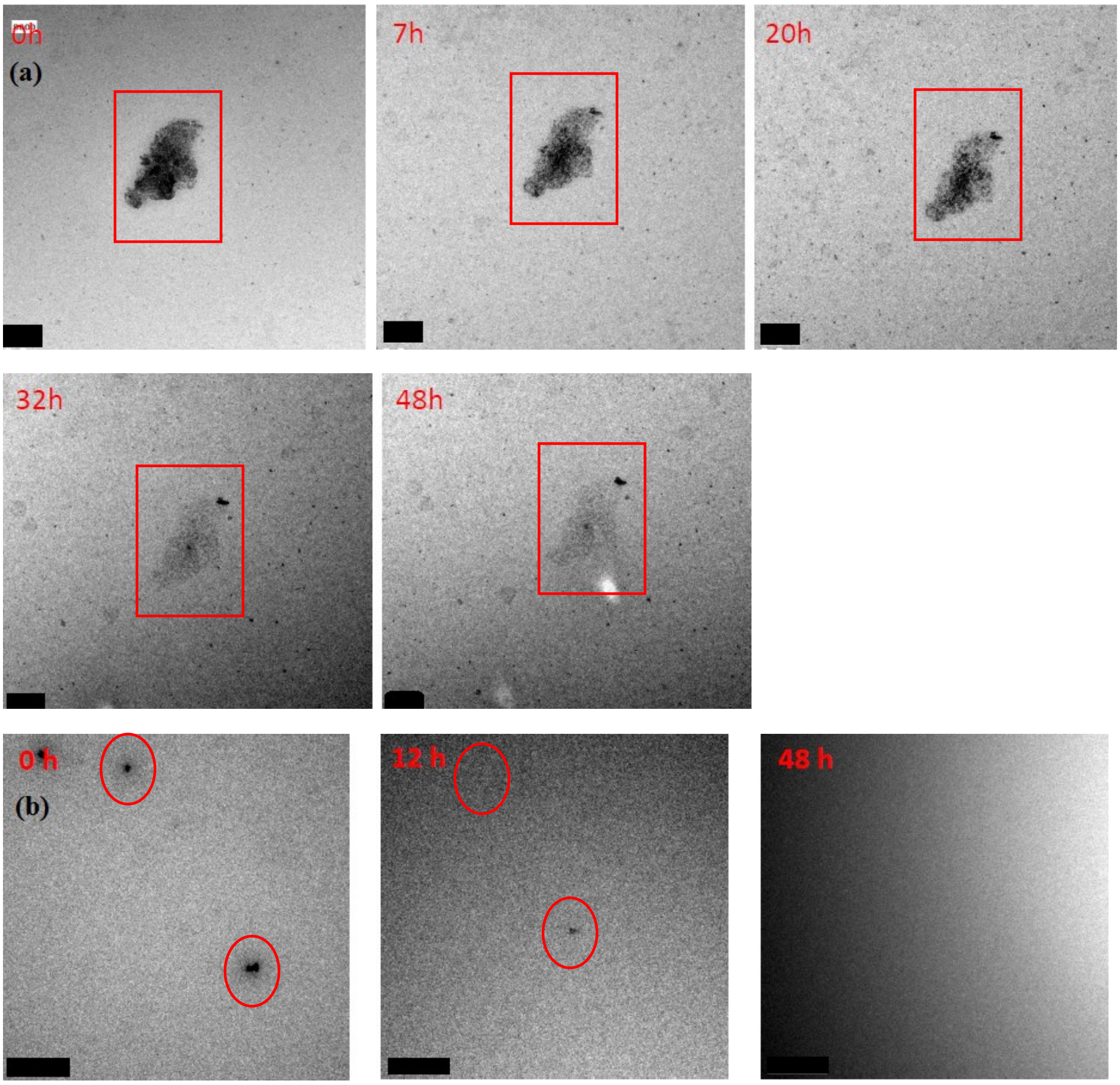

Fig. S8. *In-situ* LC-TEM dissolution of 2-line FH with10mM Fe(II)SO_4 at pH ~6. (a) Shows the dissolution under the e-beam where the solution was discharged before imaging by using the focused electron beam. The scale bar in all images is 200nm. (b) Shows the dissolution where the solution was not discharged and is measured almost without beam effects by short imaging between 1-2 seconds. The scale bar in all images is 1000nm.

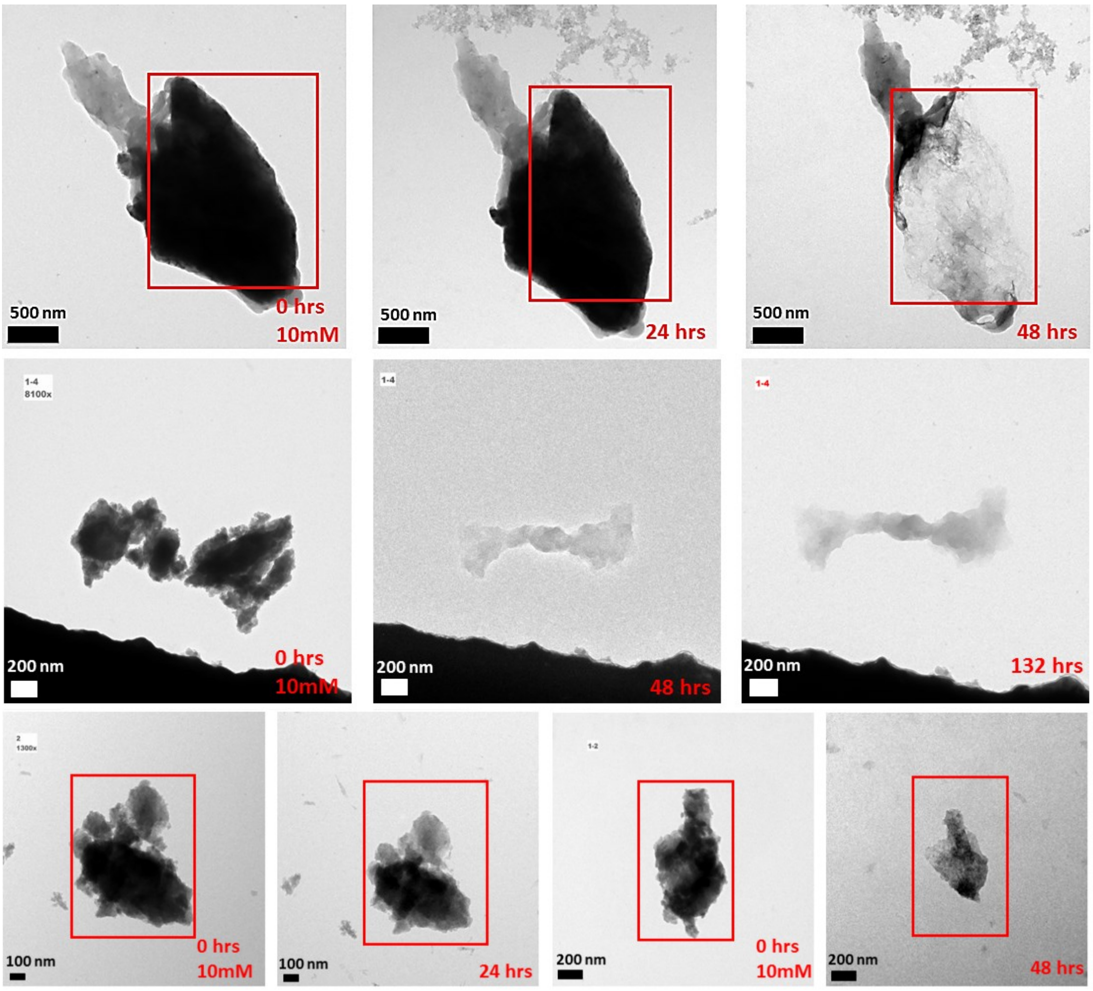
500 nm
0 hrs
10mM
500 nm
24 hrs
500 nm
48 hrs
200 nm
0 hrs
10mM
200 nm
48 hrs
200 nm
132 hrs
100 nm
0 hrs
10mM
100 nm
24 hrs
200 nm
0 hrs
10mM
200 nm
48 hrs

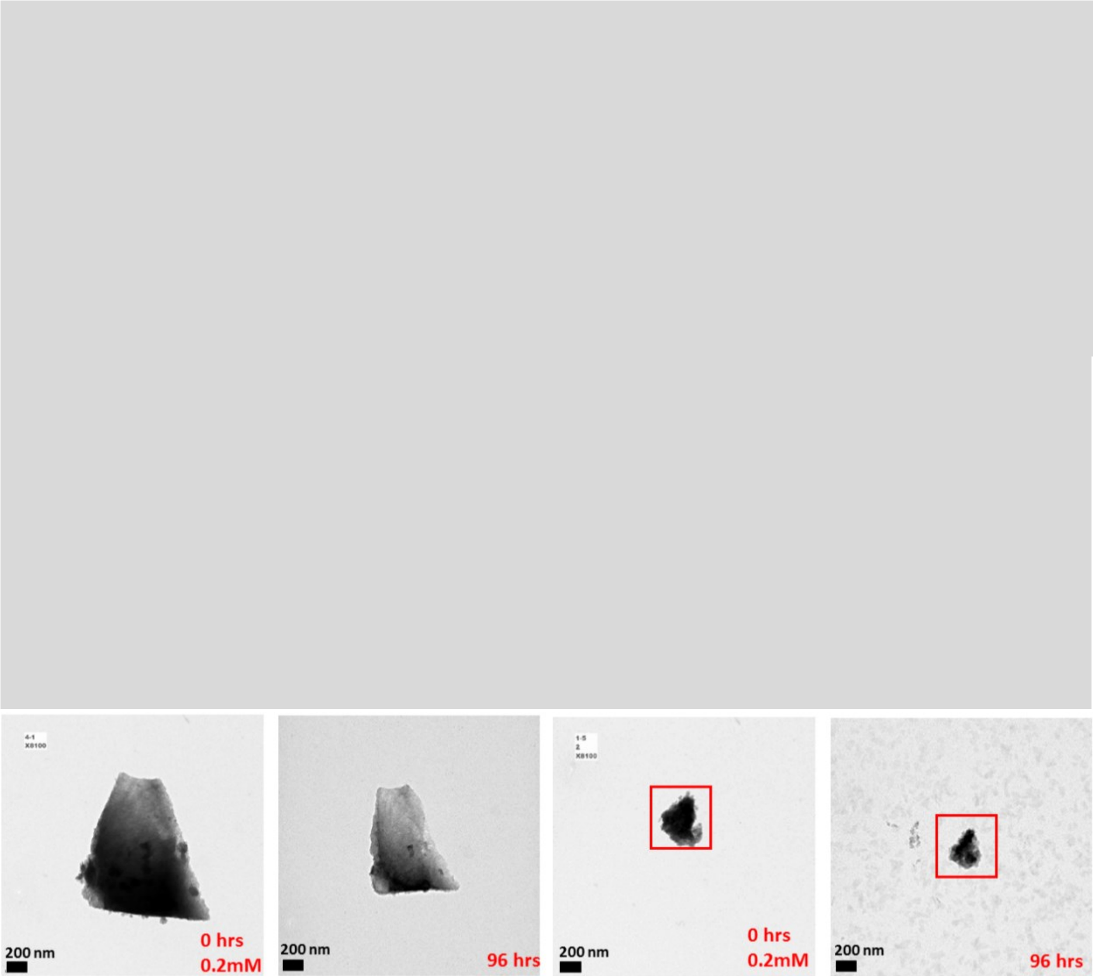

Fig. S9. Semi *in-situ* measurement of the dissolution of 2-line FH particles in 10mM and 0.2mM $Fe(II)_{(aq)}$ at pH ~6 with time. The images display the dissolution process occurring through the decrease in size and density of the particles as time proceeds.

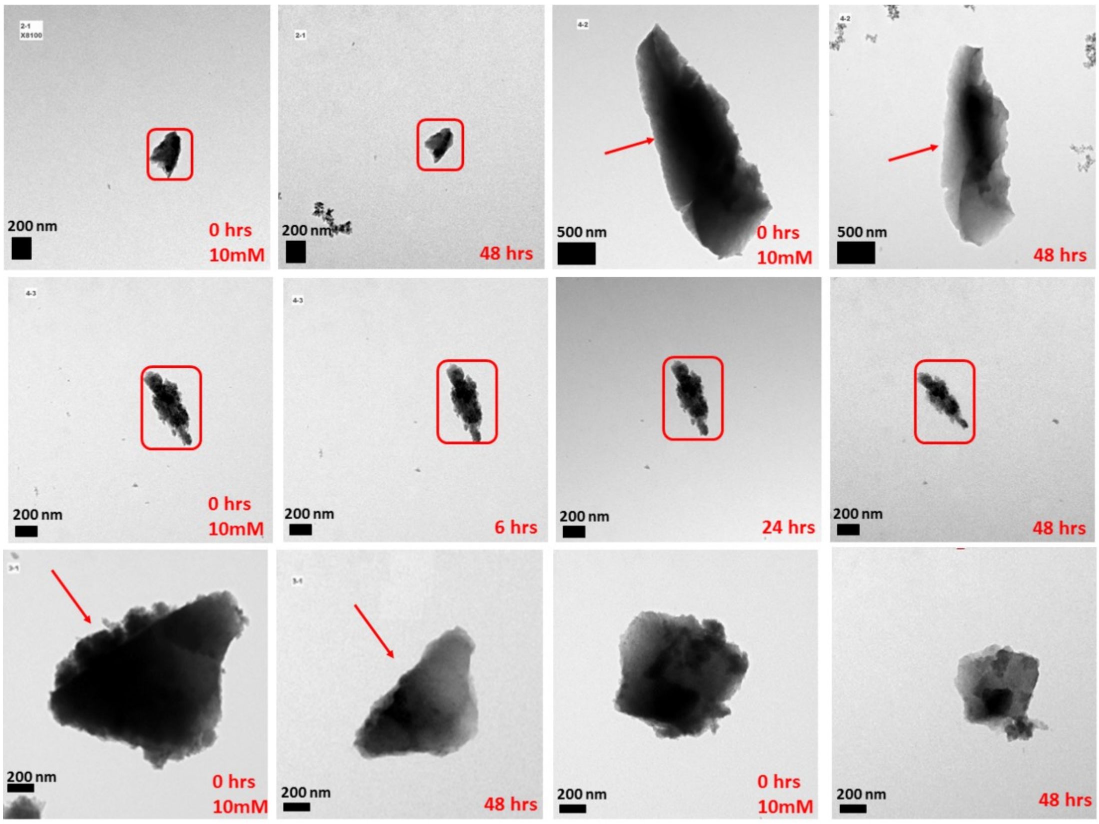
200 nm
0 hrs
10mM
200 nm
48 hrs
500 nm
0 hrs
10mM
500 nm
48 hrs
200 nm
0 hrs
10mM
200 nm
6 hrs
200 nm
24 hrs
200 nm
48 hrs
200 nm
0 hrs
10mM
200 nm
48 hrs
200 nm
0 hrs
10mM
200 nm
48 hrs

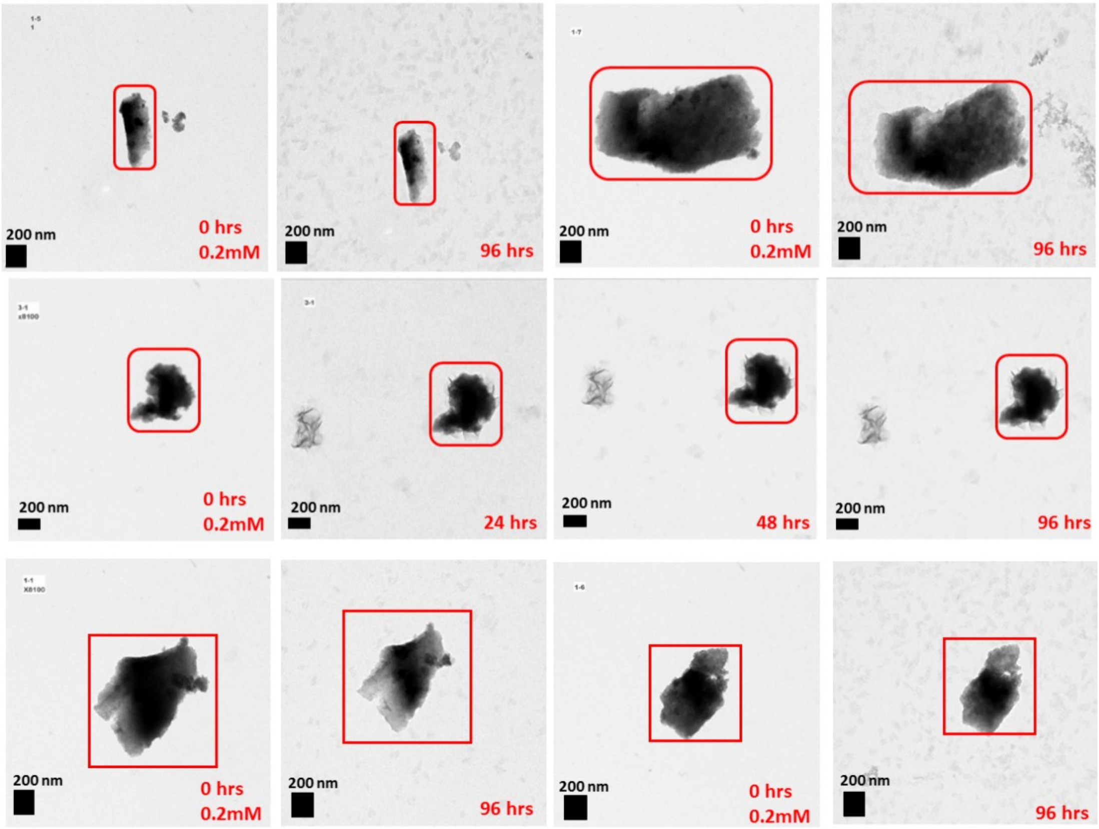
200 nm
0 hrs
0.2mM
200 nm
96 hrs
200 nm
0 hrs
0.2mM
200 nm
96 hrs
200 nm
0 hrs
0.2mM
200 nm
24 hrs
200 nm
48 hrs
200 nm
96 hrs
200 nm
0 hrs
0.2mM
200 nm
96 hrs
200 nm
0 hrs
0.2mM
200 nm
96 hrs

Fig. S10. Semi *in-situ* measurement of the lack of dissolution for 2-line FH particles in 10mM and 0.2mM $Fe(II)_{(aq)}$ at pH ~6 with time. The images display a lack of dissolution that occurs with time in both small (≤ 1µm) and large (≥ 1 µm) particles.

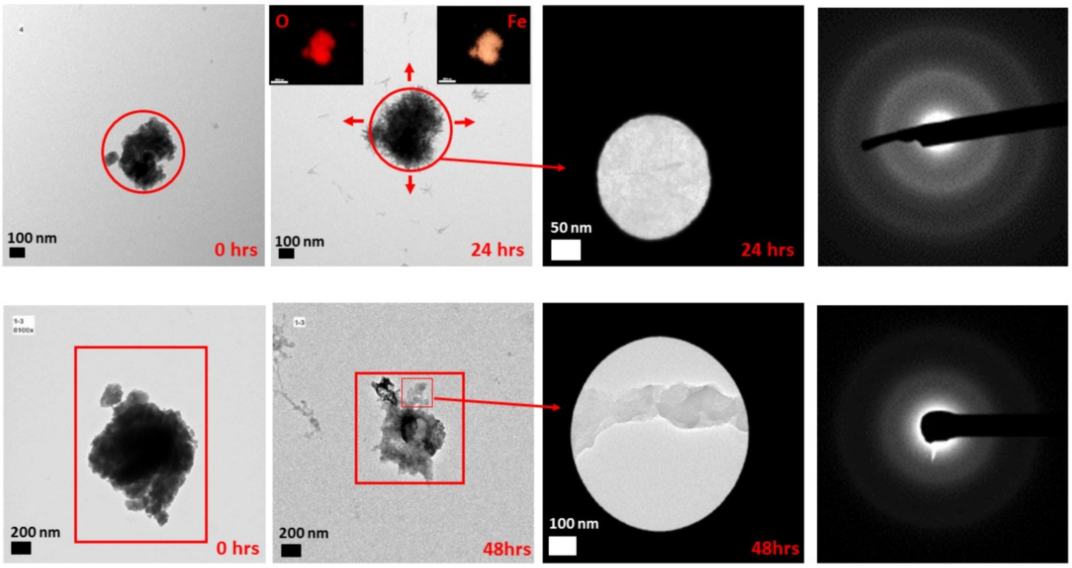

Fig. S11. 2-line FH reacted in 10mM $Fe(II)_{(aq)}$ at pH ~6 showing the amorphous layer on the surface of the reacted particle as determined by ED and the EDX of its composition. The other images show an inset close up of the amorphous surface layer and its corresponding ED pattern.

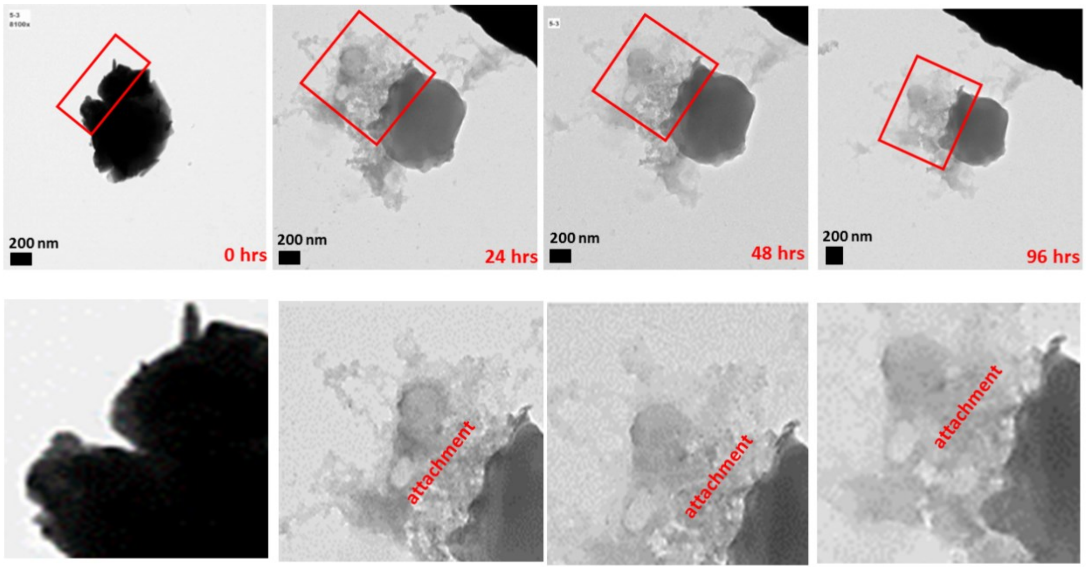

Fig. S12. Illustrates the 2-line FH reacted in 10mM $Fe(II)_{(aq)}$ at pH ~6 and various times as well as the deposition/attachment of an amorphous phase to the surface of the 2-line FH. The bottom images are a close up of the boxed regions in the images presented above at the same times.

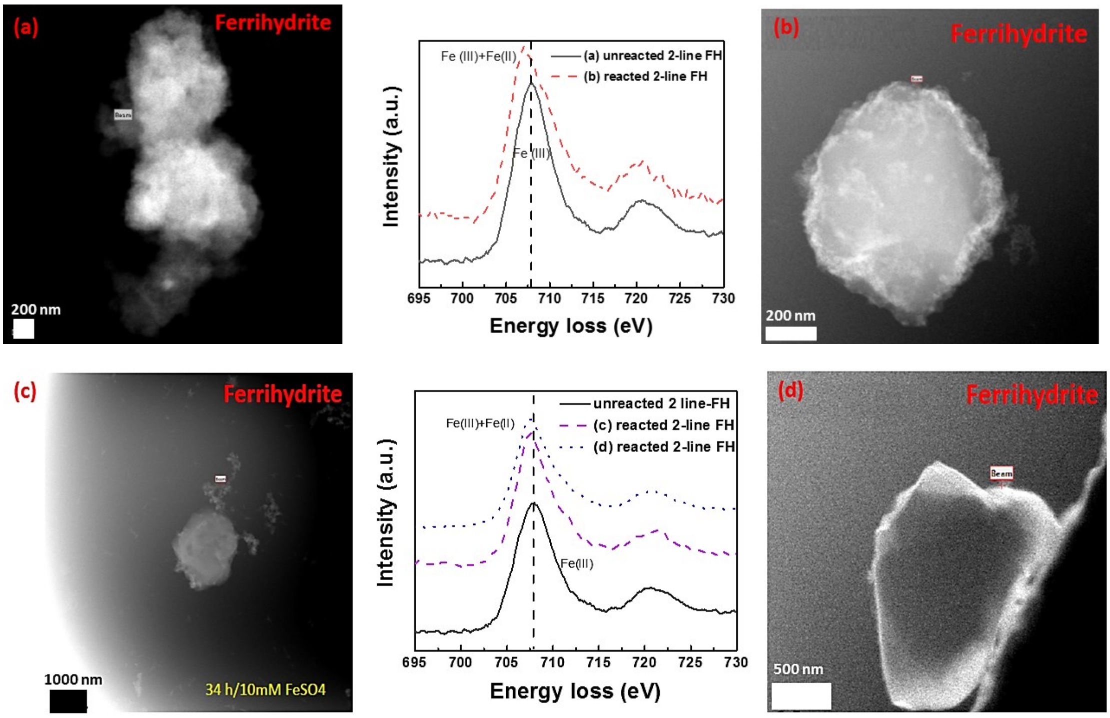

Fig. S13. Shows 2-line FH particles reacted in 10mM $Fe(II)_{(aq)}$ at pH ~6 and 34 hours. (a) The initial unreacted 2-line FH and its corresponding EELS spectrum that indicates only Fe^{3+} is observed as expected. (b) 2-line FH reacted in 10mM $Fe(II)_{(aq)}$ at pH ~6 for 34 hours showing the presence of Fe^{3+} and Fe^{2+} at the particles surface via EELS. (c) Small precipitates showing the presence of Fe^{3+} and Fe^{2+} and (d) another particle showing the presence of Fe^{3+} and Fe^{2+} at the surface. The position of the beam in the images is indicated by the small box on the particles.

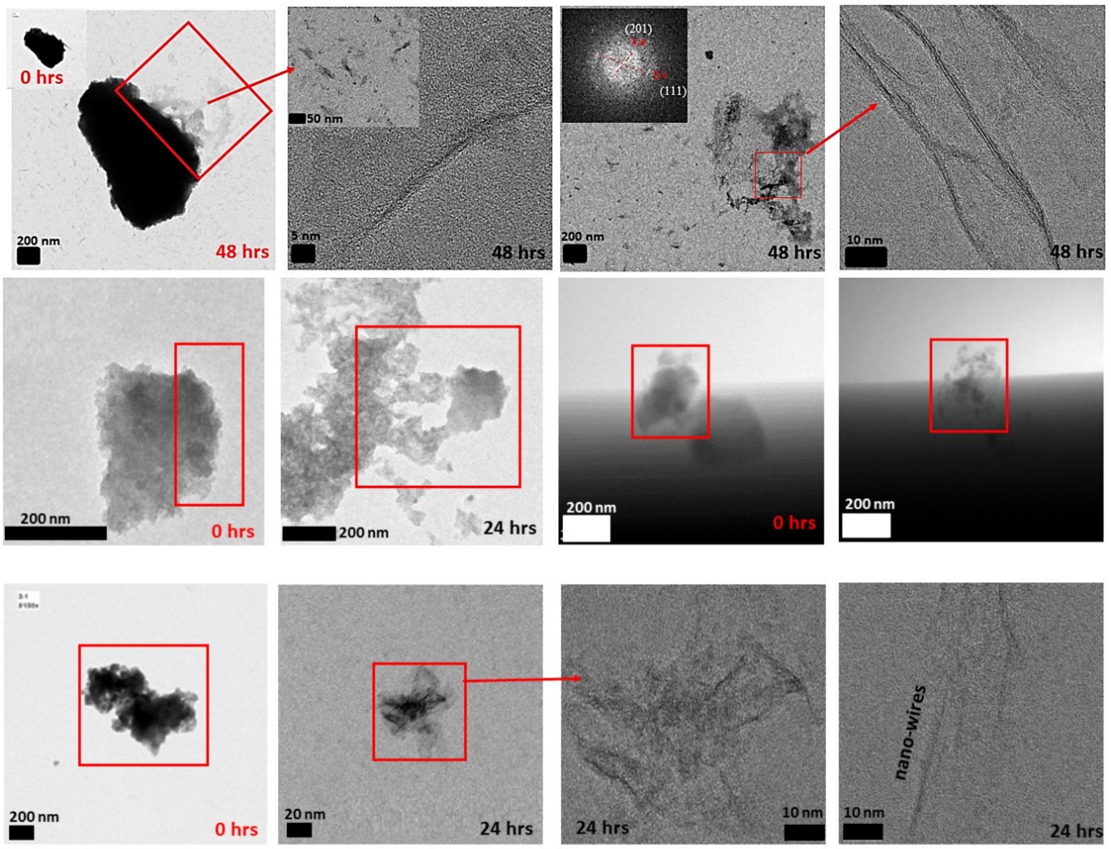

Fig. S14. Semi *in-situ* measurement of the dissolution of 2-line FH in 10mM $Fe(II)_{(aq)}$ at pH ~6 with time. The images display the dissolution process occurring through the breakage of the particles into smaller nano-particles through pores and hollow channels as well as nano-wires. Close up regions of the dissolved particles are indicated by the arrows in the images.

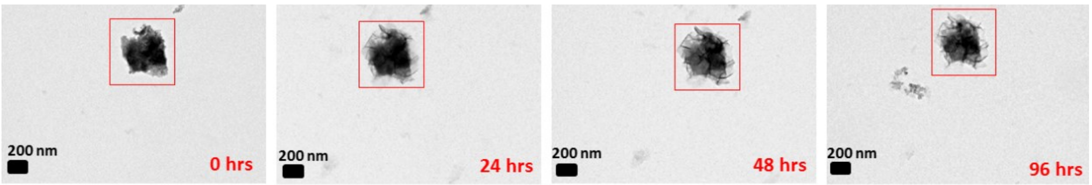

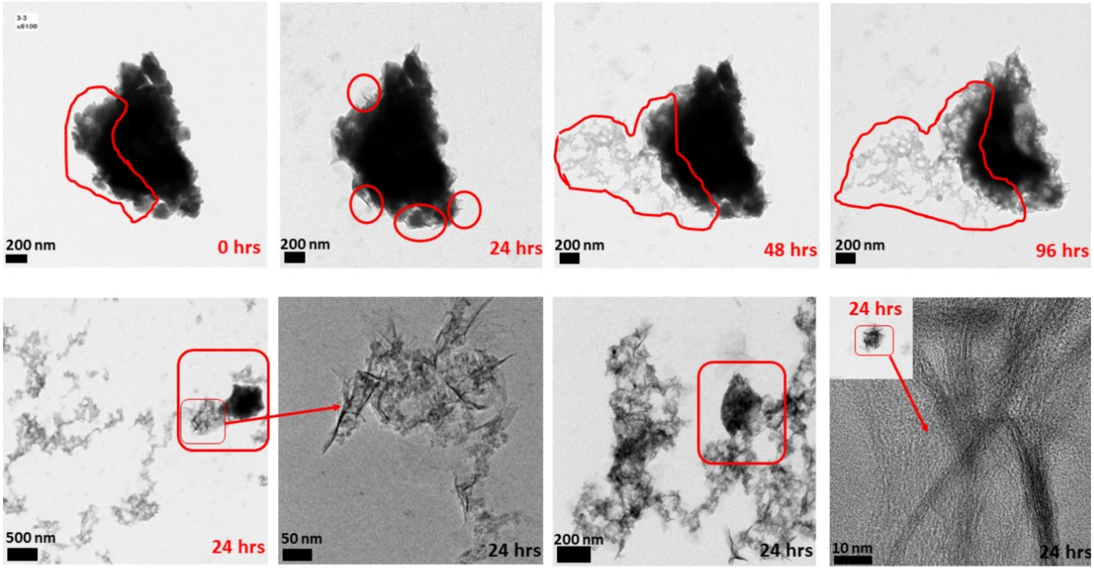

Fig. S15. Semi *in-situ* measurement of the dissolution of 2-line FH in 0.2mM $Fe(II)_{(aq)}$ at pH ~6 with time. The images display the dissolution process occurring through the breakage of the particles into smaller nano-particles as well as nano-wire whiskers coming from the surface of the reacted particles. Close up regions of the dissolved particles are indicated by the arrows in the images.

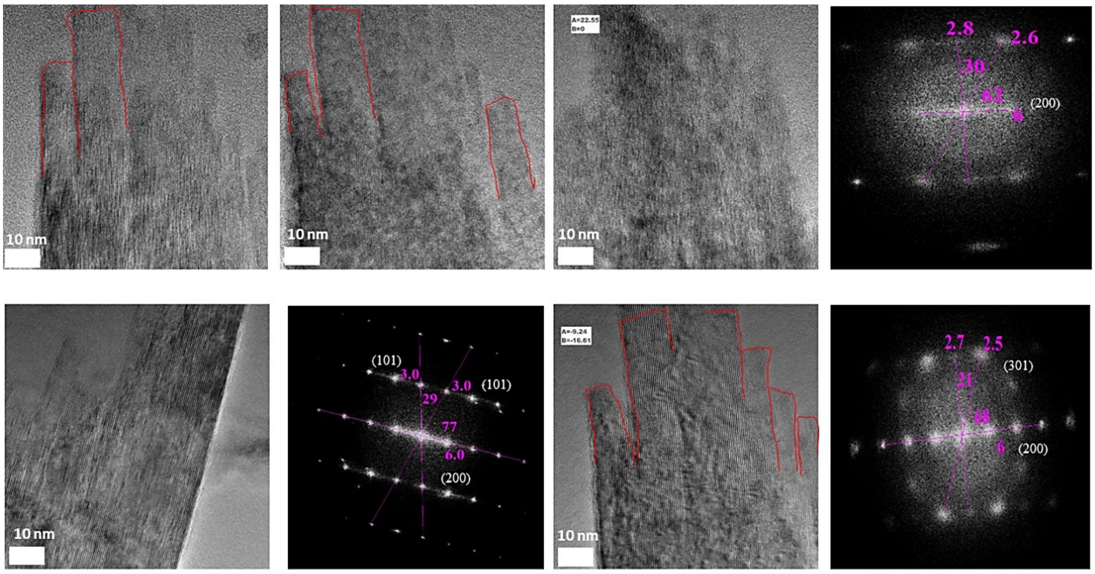

Fig. S16. Shows the formation of Lepidocrocite tabular plate-like crystals from small lamellae of elongated crystals after the reaction of 2-line FH with 10 mM $Fe(II)_{(aq)}$ at

pH ~ 6 and 6 hours. The presented ED patterns show them to correspond to the Lepidocrocite phase.

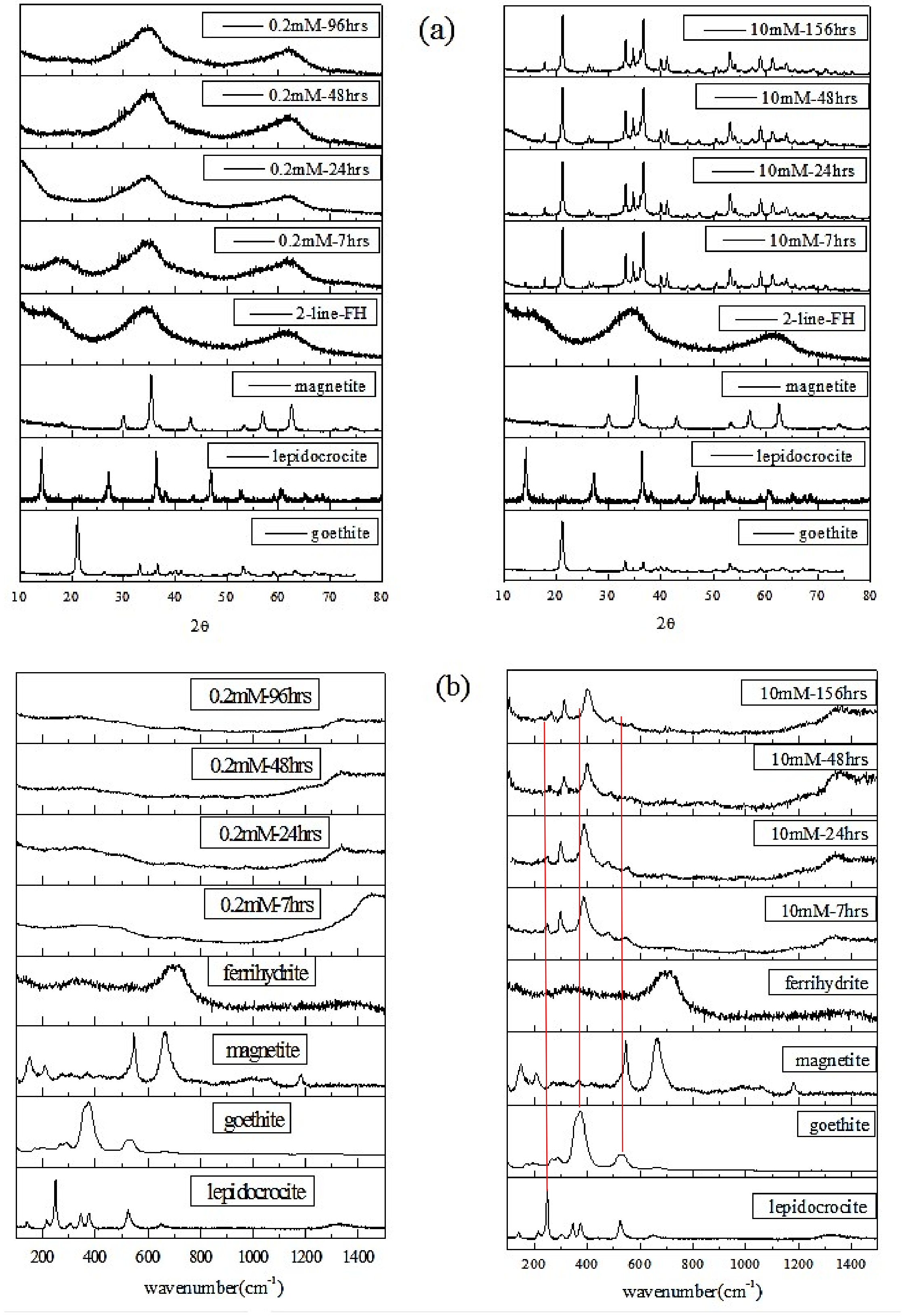
(a)
0.2mM-96hrs
0.2mM-48hrs
0.2mM-24hrs
0.2mM-7hrs
2-line-FH
magnetite
lepidocrocite
goethite
10mM-156hrs
10mM-48hrs
10mM-24hrs
10mM-7hrs
2θ
(b)
0.2mM-96hrs
0.2mM-48hrs
0.2mM-24hrs
0.2mM-7hrs
ferrihydrite
magnetite
goethite
lepidocrocite
10mM-156hrs
10mM-48hrs
10mM-24hrs
10mM-7hrs
wavenumber(cm-1)

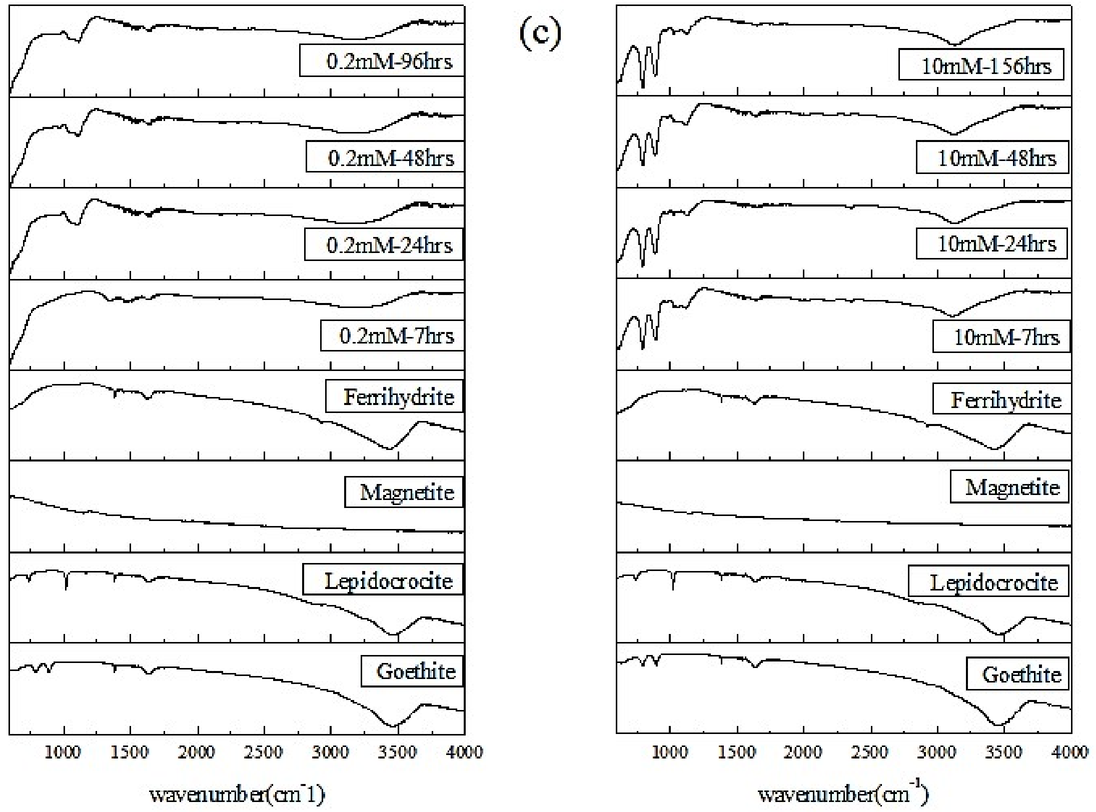

Fig. S17. Bulk (a) XRD, (b) Raman and (c) ATR-IR of 2-line FH reacted at 0.2 and 10 mM $Fe(II)_{(aq)}$ for selected amounts of time.

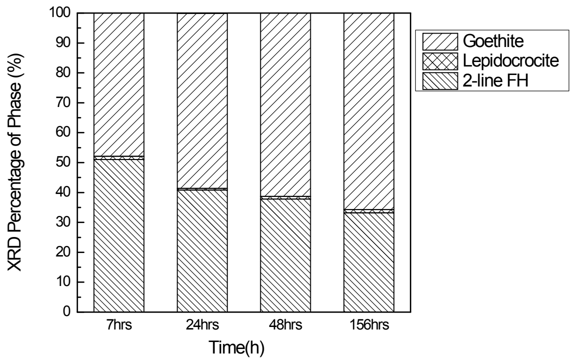

Fig. S18. Bulk XRD semi-quantitative Rietveld analysis of possible reacted phases precipitated from bulk 2-line FH reacted at 10 mM $Fe(II)_{(aq)}$ for selected amounts of time.

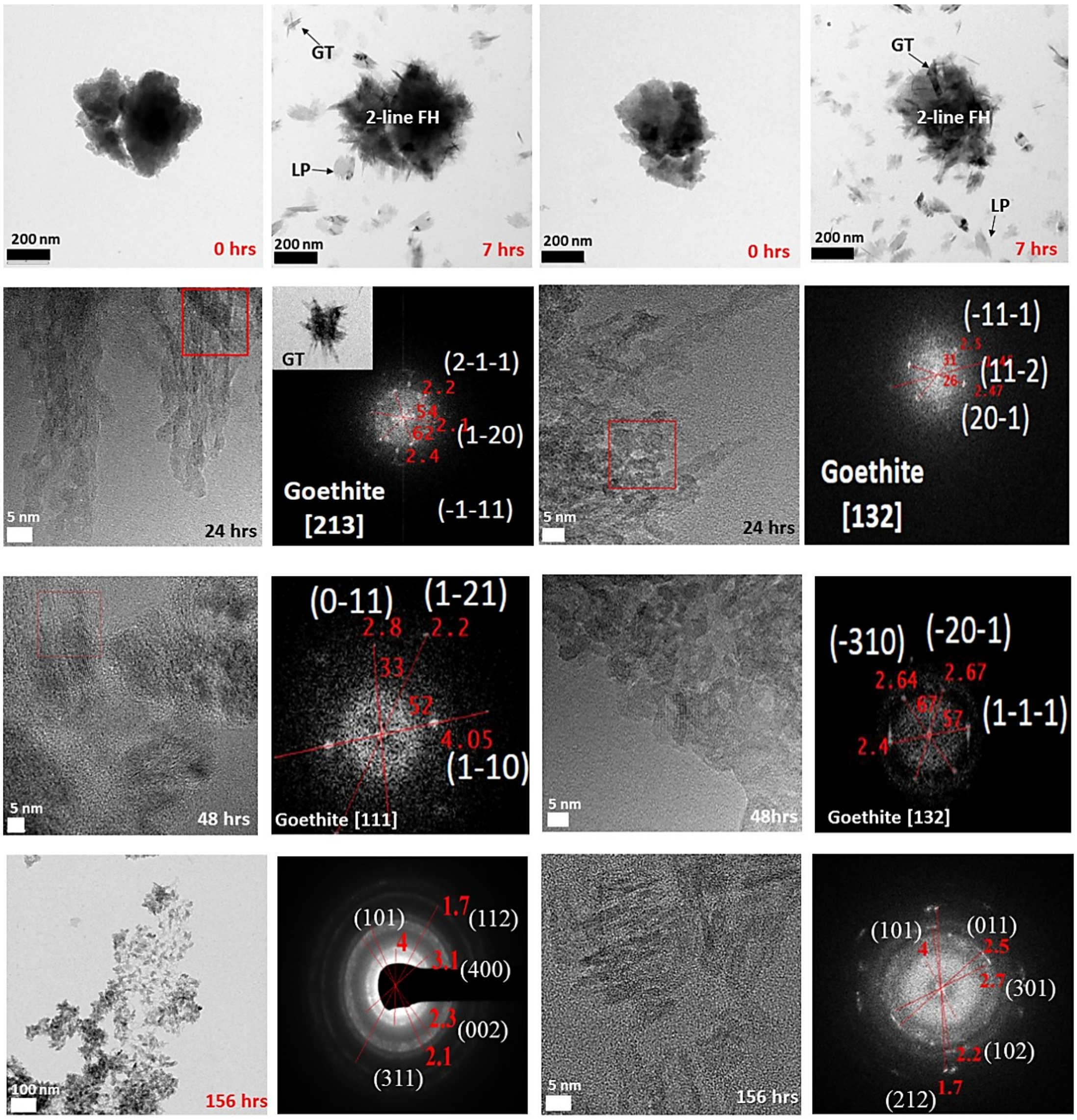

Fig. S19. Illustrates the reaction products of 2-line FH with 10mM $Fe(II)_{(aq)}$, and pH ~ 6 for various reaction times. Their ED pattern corresponding to the image beside it match with that of Goethite.

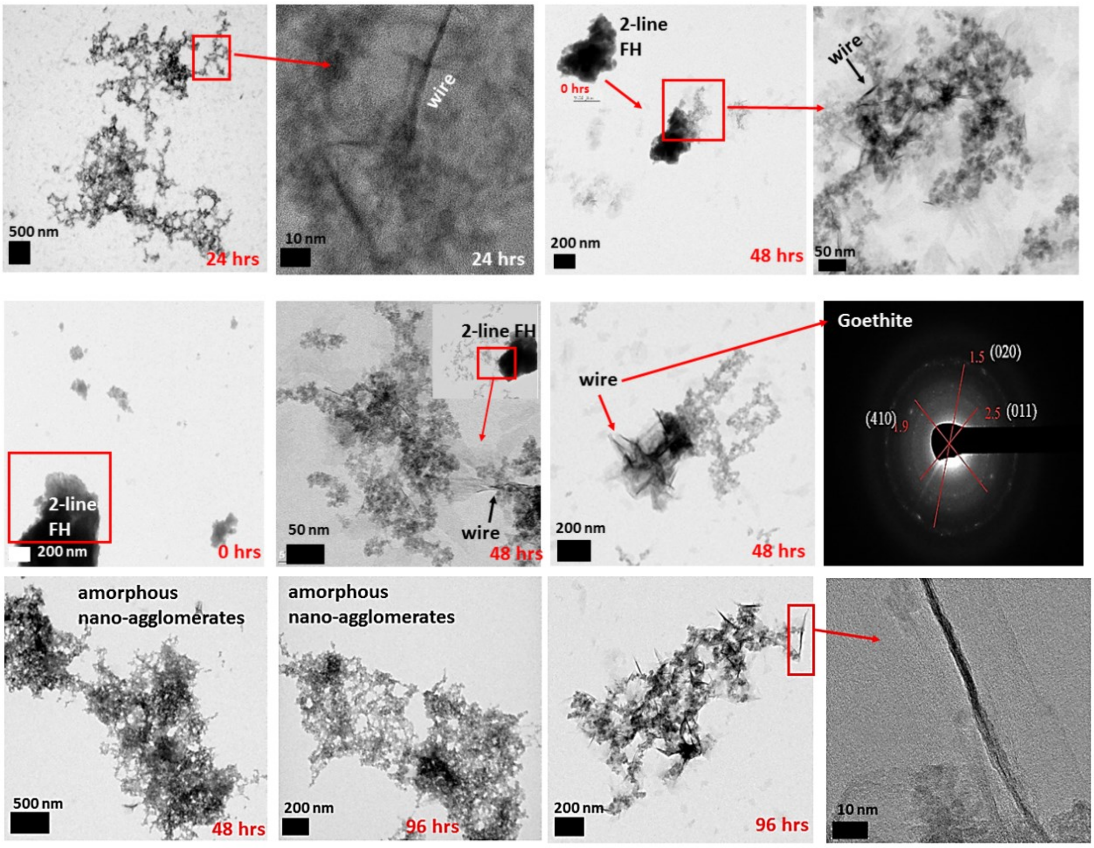

Fig. S20. Shows the reaction products of 2-line FH with 0.2 mM $Fe(II)_{(aq)}$, and pH ~ 6 for various reaction times. The ED pattern of the 48 hr particle show it to match with Goethite.

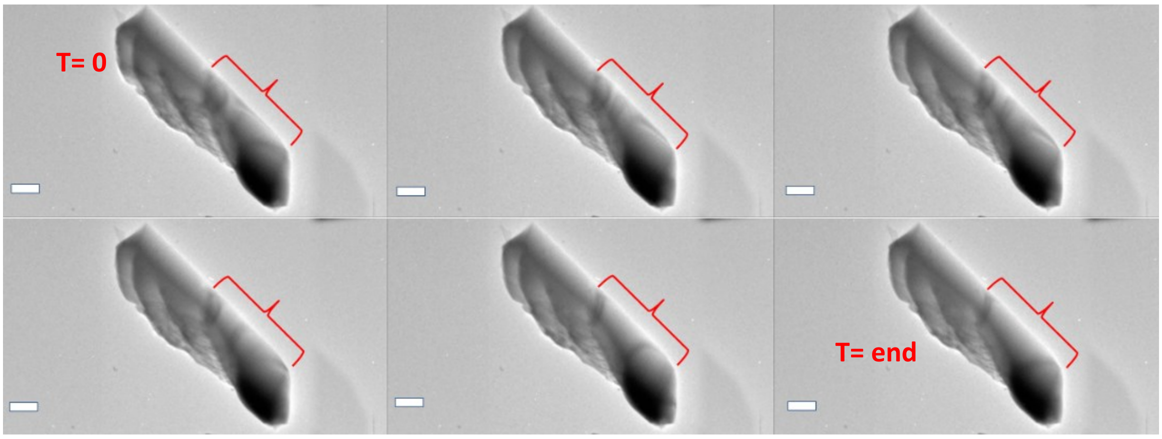

Fig. S21a. Time series I (left to right) for the *in-situ* liquid cell TEM images of the adsorption events of possible ions/molecules [10mM $Fe(II)_{(aq)}$] on a particular solid substrate [Fe(III)-Hydroxide] at a pH of 8. We can observe through the bend contours, a possible concentrated adsorbed particle region on the crystal splits into two distinct concentration bands of adsorbed particles that migrate in opposite directions along the crystal surface until they join at a higher concentration zone on the crystal surface. The scale bar on the images is 0.5 µm.

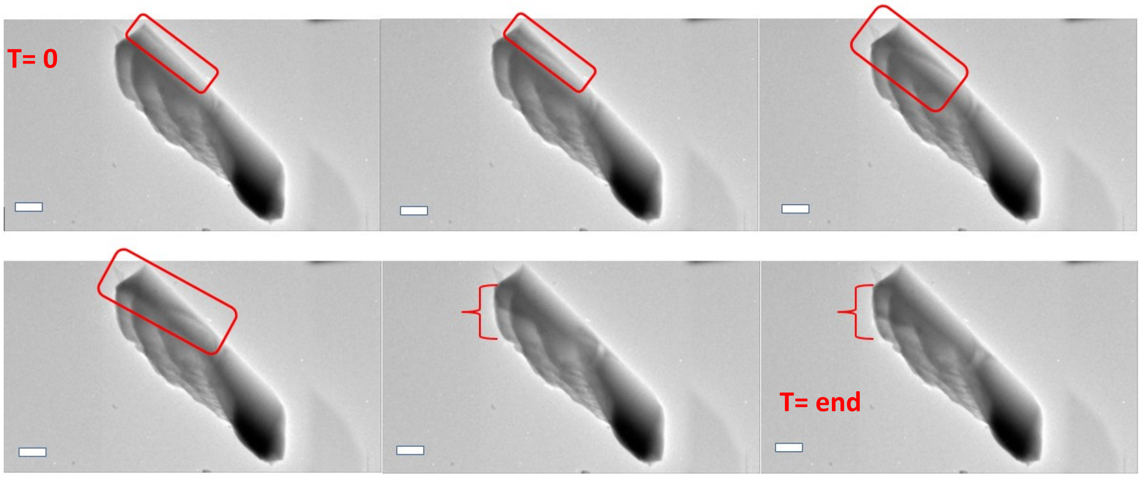

Fig. S21b. Time series II (left to right) *in-situ* liquid cell TEM images enabling the observation of the possible adsorption ("coating") reactions of ions/molecules [10mM $Fe(II)_{(aq)}$] on a particular solid substrate [Fe(III)-Hydroxide] at a set pH of 8. We can observe the discrete adsorption/coating of a material [likely in the form of $Fe(II)_{(aq)}$] on the Fe(III)-Hydroxide crystal surface. This adsorption/coating event is expressed in the TEM image/video via the particle becoming darker in contrast (through bend contours) as the crystal is coated/adsorbed by the ions/molecules in solution. The scale bar is 0.5 µm in all the images.
